# The Whereabouts of Flower Visitors: Contrasting Land-Use Preferences Revealed by a Country-Wide Survey Based on Citizen Science

**DOI:** 10.1371/journal.pone.0045822

**Published:** 2012-09-19

**Authors:** Nicolas Deguines, Romain Julliard, Mathieu de Flores, Colin Fontaine

**Affiliations:** 1 MNHN-CNRS-UPMC, UMR 7204 CERSP, Muséum national d'Histoire naturelle, Paris, France; 2 Office Pour les Insectes et leur Environnement, Guyancourt, France; University of Northampton, United Kingdom

## Abstract

**Background:**

In the past decade, accumulating evidence of pollinator decline has raised concerns regarding the functioning of terrestrial ecosystems and the sustainability of crop production. Although land-use changes have been advanced as the major causes, the affinities of most wild pollinators with the main land-use types remain unknown. Filling this gap in our knowledge is a prerequisite to improving conservation and management programmes.

**Methodology/Principal Findings:**

We estimated the affinity of flower visitors with urban, agricultural and natural land-uses using data from a country-wide scale monitoring scheme based on citizen science (Spipoll). We tested whether the affinities differed among insect orders and according to insect frequency (frequent or infrequent). Our results indicate that the affinities with the three land-use types differed among insect orders. Apart from Hymenopterans, which appeared tolerant to the different land-uses, all flower visitors presented a negative affinity with urban areas and a positive affinity with agricultural and natural areas. Additionally, infrequent taxa displayed a lower affinity with urban areas and a higher affinity with natural areas than did frequent taxa. Within frequent taxa, Hymenoptera and Coleoptera included specialists of the three land-use types whereas Diptera and Lepidoptera contained specialists of all but urban areas.

**Conclusions/Significance:**

Our approach allowed the first standardised evaluation of the affinity of flower visitors with the main land-use types across a broad taxonomical range and a wide geographic scope. Our results suggest that the most detrimental land-use change for flower visitor communities is urbanisation. Moreover, our findings highlight the fact that agricultural areas have the potential to host highly diverse pollinator communities. We suggest that policy makers should, therefore, focus on the implementation of pollinator-friendly practices in agricultural lands. This may be a win-win strategy, as both biodiversity and crop production may benefit from healthier communities of flower visitors in these areas.

## Introduction

Animal pollination is a key process in the functioning of terrestrial ecosystems, as is shown by the fact that the reproduction of 87.5% of flowering plants depends on it [1]. The production of about 70% of main crops also relies on pollinators [2] and the value of this pollination service has been estimated worldwide at €153 billion for 2005 alone [3]. Biodiversity loss is continuing [4], including for insect pollinators [5], [6], and their decline raised concerns about the potential consequences to both natural and agro-ecosystems [7]. Indeed, pollinator diversity is crucial for the persistence of plant communities [8] and it both increases and stabilises agricultural production [9]. Recognising the utmost value of pollination and the current threats on it, policy-makers have supported the cause of pollinators [10], [11].

Whereas the geographical extent and the current rate of decline of most pollinators remain unknown (but see [5], [6], [12], [13]), the causes have been widely investigated, and habitat degradation has been put forward as the most important driver of pollinator loss [14]. Habitat degradation occurs within the three broad types of land-use – i.e. the urban, agricultural and natural land-use types – and its effects on pollinator diversity are relatively well known. Urbanisation, the expansion and densification of urban areas, mostly has a negative effect on pollinator richness although results may vary at intermediate levels of urbanisation [15]. Agricultural intensification has been shown to decrease bee, hoverfly and butterfly diversity [9], [16], [17]. It remains uncertain whether habitat fragmentation *per se* [18] is a cause of pollinator loss as changes in species composition do not always turn into diversity loss [19], [20]. In contrast, the comparative suitability of these different land-use types for pollinators has been poorly investigated. Additionally, comparisons between groups of pollinators are unreliable, as studies have often focused on restricted groups of pollinators and used different sampling and habitat characterisation methods. As a consequence, a comprehensive picture of pollinator response to land-use changes remains missing [21].

**Figure 1:**
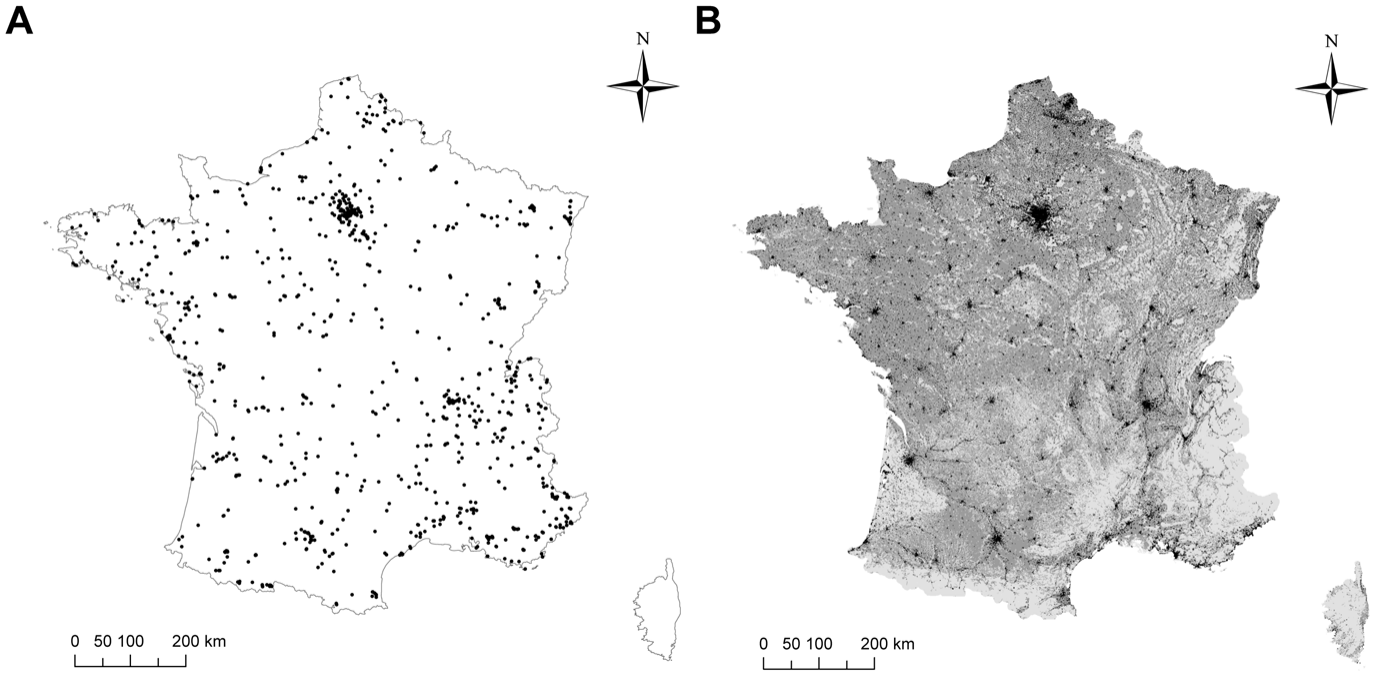
Sampling sites and land-use types spatial distributions. The spatial distribution of (A) the 2131 collections (i.e. sampling sites) analysed in 2010, and (B) the urban, agricultural and natural land-use types in France, represented in dark, medium and light grey, respectively.

These gaps in our knowledge highlight the absence of indicators to characterise the current status and trends of pollinators across a broad geographical range. Policy-makers need such information to develop most effective management for different pollinators. Until now, existing biodiversity indicators have been biased towards birds and mammals [22], and policy-makers lack information to meet the challenge of preserving pollination in ecosystems. A major difficulty in producing such indicators for pollinators is their tremendous diversity. Pollinators are not restricted to bees or hoverflies but also include frequent flower visitors, such as beetles or other flies, particularly in natural plant communities [23], [24]. As a matter of fact, there are many examples of flower visitors having been unexpectedly identified as primary pollinators of plants [25]–[27]. Therefore, to provide informative indicators of pollinators, it is necessary to enlarge the geographical scale of sampling, both within and between land-use types, and to broaden the taxonomic scope to all flower visitors.

**Table 1:** Number of insect taxa recorded among orders and by taxonomic resolution.

Taxonomic resolution	Coleoptera		Diptera		Hymenoptera		Lepidoptera	
	Freq	Infreq	Freq	Infreq	Freq	Infreq	Freq	Infreq
a whole family	2	5	2	5	1	2	0	3
	(111)	(31)	(94)	(30)	(141)	(18)	(0)	(23)
several genera within a family	1	5	2	3	3	3	1	3
	(63)	(8)	(101)	(32)	(583)	(11)	(44)	(16)
species from different genera	7	12	5	3	5	12	6	6
	(433)	(83)	(630)	(34)	(444)	(83)	(309)	(44)
a genus	1	7	3	14	0	3	0	7
	(19)	(65)	(250)	(108)	(0)	(45)	(0)	(71)
species from a genus	0	7	1	2	3	7	3	10
	(0)	(52)	(30)	(8)	(1055)	(93)	(239)	(82)
a single species	4	27	5	18	2	10	3	49
	(212)	(102)	(689)	(121)	(641)	(54)	(142)	(275)
total	15	63	18	45	14	37	13	78
	(838)	(341)	(1794)	(333)	(2864)	(304)	(734)	(511)

The distributions of frequent (Freq) and infrequent (Infreq) taxa among orders and by taxonomic resolution. The corresponding numbers of observations (number of pictures) are in brackets.

To fulfil these objectives, we started a monitoring scheme of all flower visitors across France (the Photographic Survey of Flower Visitors, hereafter Spipoll) that relies on citizen science, i.e. ‘*the involvement of volunteers in research*’ [28]. In various fields of ecology, scientific outcomes based on citizen science are flourishing [29]–[34], and this approach seemed particularly appropriate to provide the Spipoll with the numerous data needed at a country-wide scale. Using standardised data gathered by volunteers, we developed an index of affinity that estimates the extent to which a flower visitor is observed more (or less) often in a particular land-use type than expected given its frequency of occurrence and our sampling of the land-use types. In the present study, we compare the affinities of flower-visitors with the three broad types of land-use found in France, i.e. urban, agricultural and natural areas. Specifically, we ask whether the affinities of flower visitors with the three types of land-use are similar, how communities of flower visitors may respond to land-use changes and what the potential implications for the management of flower visitors are. We answer these questions in relation to the four main orders of flower visitors (Coleoptera, Diptera, Hymenoptera and Lepidoptera) and distinguishing between frequent taxa and infrequent taxa.

**Figure 2:**
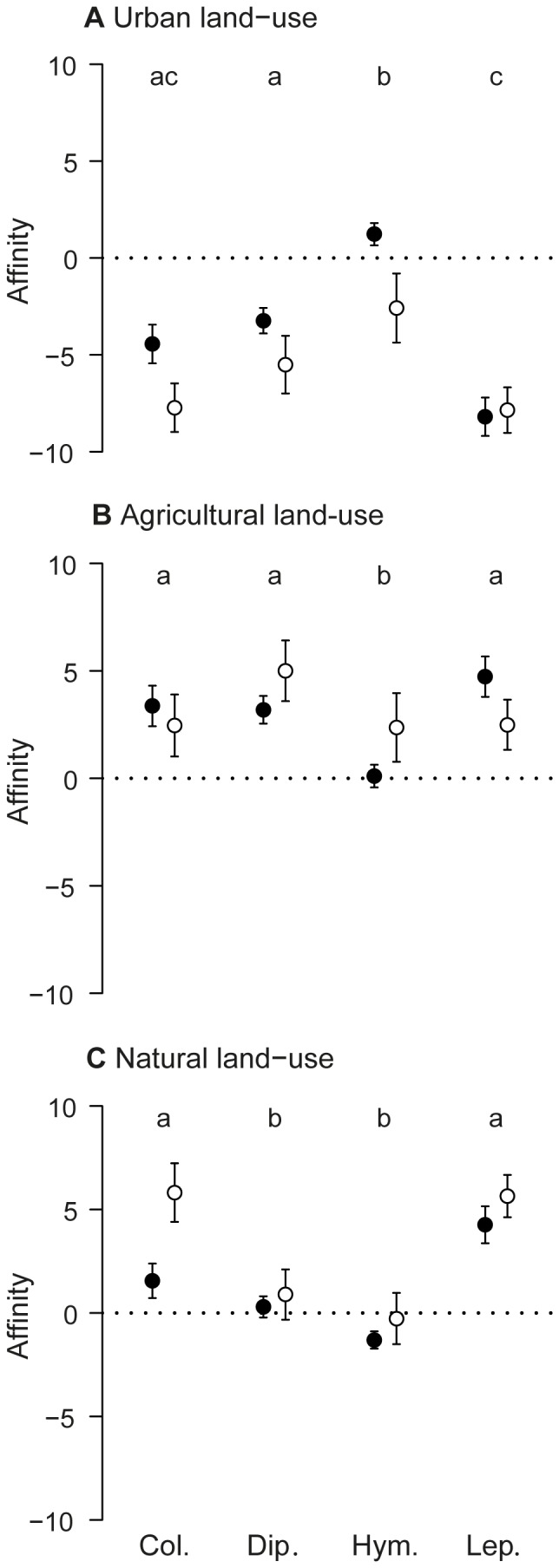
Affinities of the four orders of flower visitors with the three land-use types. The affinities with the (A) urban, (B) agricultural and (C) natural land-use of the frequent (black dots) and infrequent (white dots) taxa within the four orders (Col., Dip., Hym. and Lep., standing for Coleoptera, Diptera, Hymenoptera and Lepidoptera, respectively). Bars are standard errors and letters indicate differences among orders according to the Tukey's honest significance tests performed.

**Table 2:** Type-III ANOVA results for the relative land-use indexes.

Dependent variables	Effect	Df	F value	Pr(>F)
Relative urban land-use index	Order	3	27.627	<0.001***
	Frequency	1	6.177	0.013*
Relative agricultural land-use index	Order	3	8.397	<0.001***
Relative natural land-use index	Order	3	18.495	<0.001***
	Frequency	1	6.343	0.012*

Type-III ANOVA results for the three relative land-use indexes. The results shown are from the minimum adequate models. The ‘F-value’ is the value from F distribution.

## Materials and Methods

### Sampling protocol

The Spipoll protocol is ideally suited to the abilities of a broad range of volunteers because it is both simple and playful, yet standardised, and does not require knowledge of either insects or plants. Basically, the aim of the protocol is to compile a photographic collection of insects interacting with a plant species at a given place and time.

**Figure 3:**
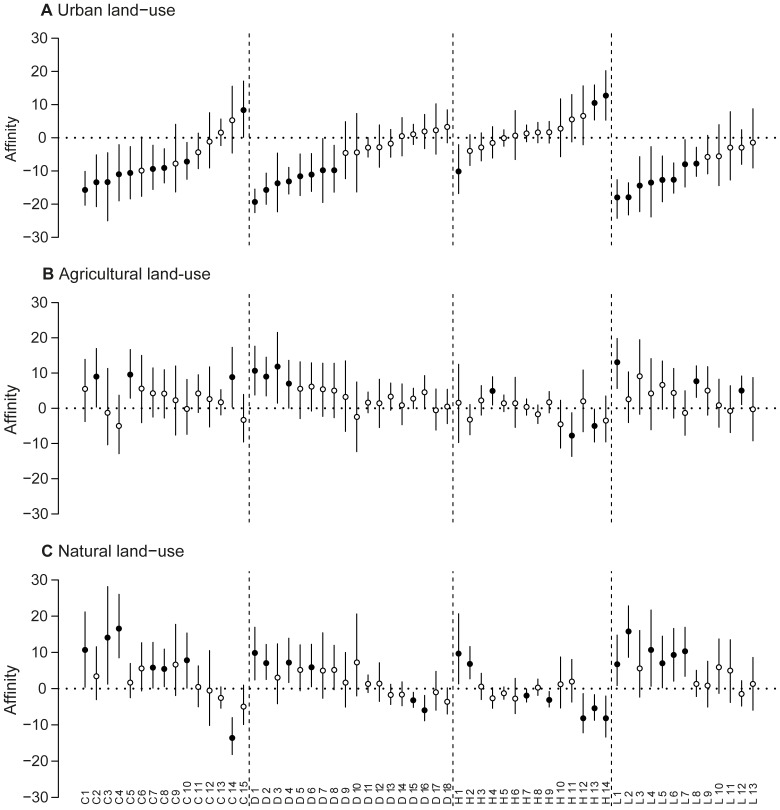
Affinities of the 60 frequent taxa with the three land-use types. Affinities of the 60 frequent taxa with (A) urban, (B) agricultural and (C) natural land-use. Letters and numbers at the bottom are taxa' identity: C 1–15, D 1–18, H 1–14 and L 1–13 are Coleopterans, Dipterans, Hymenopterans and Lepidopterans, respectively (Table S1). Taxa are sorted by order and increasing affinity with urban land-use. Bars are 95% BCa confidence intervals. Black dots are the specialist and avoider taxa (affinity significantly different from zero) and white dots are tolerant taxa (affinity not significantly different from zero).

Data collection is the first step in volunteer participation. Wherever in mainland France, observers were asked to choose a flowering plant species, this could include several individuals within a 10 m^2^ area, and to take two pictures: one of the flowers and one of the close environment. Then, they were asked to photograph all insects either feeding or landing on the flowers over a 20-minute period. Another version of the protocol (‘Long protocol’) allows longer but recorded duration of observation time. Data from both protocols were used in the present paper, as it allowed more precise estimation of affinity of taxa while our results are robust to the removal of data from the Long protocol.

The second step is to sort insects' pictures into taxa (also known as morphospecies or parataxonomic units [35]), each being a group of species differing from all the other groups in any external features that can be seen on pictures of live un-captured arthropods.

The third step consists in uploading one picture per insect taxon and the pictures of the flowers and their close environment onto the Spipoll's website. Observers were asked to fill in information relative to their observations regarding the date, precise location, duration, climatic conditions and habitat characteristics.

Finally, the last step is to identify the flower and insects. To do so, two computer-aided identification tools (CAITs) were developed, one for plants and one for insects.

### Computer-aided identification tools

A major challenge in monitoring flower visitor is in their identification, the difficulty of which is inversely proportional to the number of specialists able to identify them [36]. The CAITs were developed to identify plant and insect taxa relying only on the pictures taken in the field. They were designed so that volunteers without even the slightest knowledge of plants or insects could accomplish this task.

We used the Delta Editor programme [37], [38] to describe 333 plant taxa and 556 insect taxa. The plant taxa included a wide array of wild species and some ornamental species and the insect taxa encompassed the whole arthropod fauna that can be found on flowers in France. As identification relies on the pictures taken during the data collection, only descriptors that could be seen on pictures were used. Consequently, the taxonomic resolution of insect taxa ranged from ‘Species from different families’ (23 taxa) to ‘a species’ (280 taxa) (see Figure S1). The genus was known for 397 insect taxa (71%). The following analysis includes only taxa whose taxonomy was resolved at least to the family level. Plant taxonomic resolution ranged from ‘Several genera’ (within one family) (12 taxa) to ‘a species’ (121 taxa) (Figure S2). The genus was known for 321 plant taxa (96%).

It must be emphasised here that the use of the CAITs ensures a consistent sorting of taxa across all citizen-scientists. In other words, taxa are not defined in various ways according to each volunteer's ability to detect differences in external features, as can be the case in other methodologies relying on taxa [39].

Practically, the CAITs are online identification keys and consist in simple interfaces used by participants to identify by themselves their plant and insects. In comparison with single-access (dichotomous) identification keys, the CAITs are multi-access keys that allow their users to choose the descriptors they want to answer and ignore those they cannot. Both the plant and insect CAITs are illustrated with clear pictures and texts. They are freely available from http://www.spipoll.org/identification/flore.php and http://www.spipoll.org/identification/insectes.php, respectively.

### Data validation

We carried out the analyses on an entirely validated dataset. Entomologists from the Opie (Office for insects and their environment, an entomological NGO) reviewed the 13161 insect pictures gathered in 2010 and corrected their identification when necessary (34% of insects' identification). We removed from the analyses pictures that could not be attributed to a single taxon with certainty and whole collections that did not follow the protocol (e.g. mislocalised in water bodies, pictures taken on different plant species). Botanists from the French National Museum of Natural History validated the 2252 flower pictures.

### Land-use classification

We used the first level of the Corine Land Cover 2006 database [40] to define the three land-use types studied here. Urban and agricultural land-use types were respectively defined as the “Artificial surfaces” (which includes green urban areas such as urban parks) and the “Agricultural areas”. The natural land-use type was obtained by lumping the categories “Forests and semi natural areas” and “Wetlands”. The “water bodies” were left out. With these definitions, the three types of land-use included different habitats that vary substantially within any one type. For instance, a parking lot differs considerably from an urban park, and a highly intensive field also differs considerably from an extensive pasture. Our interest, however, lay in comparing the affinity of flower visitors with the three broad types of land-use acknowledging their inner heterogeneity.

### Land-use characterisation at sampling sites

The three land-use types are heterogeneously distributed in France, and our sampling was biased toward human-dense areas (Figure 1). To control for this heterogeneity, we characterised the landscape surrounding each collection locally relatively to the one sampled regionally. For a given land-use type and collection, we calculated a relative land-use index as the proportion of this land-use in a 1-km radius buffer around the collection minus the mean of the proportions of this land-use in a 1-km radius buffer around all the collections found within 100 km of the focal collection (equation 1).
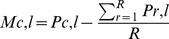
(1)where: *P_c,l_* (*P_r,l_*) is the proportion of the land-use type *l* within 1 km of collection *c* (*r*); *R* is the number of collections within 100 km of collection *c*; *M_c,l_* is the relative land-use type *l* index of collection *c*.

A high relative land-use index for a given land-use characterises a collection that has a higher local proportion of this land-use compared to the collections present regionally. For every collection, a minimum of 30 collections within 100 km was set for calculating their mean. We therefore discarded 28 collections that did not reach this threshold and based our analyses on 2131 collections.

### Affinity with the three land-use types

For a given taxon, or group of taxa, and a given land-use type, the affinity measures the extent to which individuals of this taxon have been observed in collections which had differing local proportion of this land-use compared to the collections present regionally.

We calculated the affinity of a taxon with a land-use type as the mean of the relative land-use index of the collections where this taxon had been recorded (equation 2).
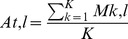
(2)where: *K* is the number of collections where taxon *t* was recorded; *A_t,l_* is the affinity of taxon *t* with the land-use type *l*.

A taxon with a positive affinity with a given land-use type reflected a preference for this land-use type. Conversely, a negative affinity meant avoidance of this land-use type.

### Frequent and infrequent insect taxa

We classified each observed taxon as either “infrequent” or “frequent”. Controlling for the sampling bias stated above, we considered a taxon to be “infrequent” if it was present in less than 2% of the collections made in each land-use type (we used the land-use type at the sampling point to count the number of collections per land-use type). On the other hand, a taxon present in more than 2% of the collections made in at least one of the three land-use types was classified as “frequent”.

### Differences of affinity among orders

To test whether the affinities with the three types of land-use differed among the four orders of flower visitors and between the frequency class, we analysed jointly the three relative land-use indexes (one for each land-use type), as they were, by construction, dependent (a collection cannot have higher local proportions of the three land-use types than do collections present regionally). We performed a type-III multivariate analysis of variance (MANOVA) that included as explanatory variables the order of taxa (factor with four levels: Coleoptera, Diptera, Hymenoptera and Lepidoptera), the frequency of taxa (factor with two levels: infrequent and frequent) and the interaction between both. As this analysis revealed significant effects of the explanatory variables (Table S3), we then performed three separate type-III univariate analyses of variance (ANOVA) with similar model structure to further investigate these effects on each of the relative land-use indexes [41]. We then reduced each model to the minimum adequate model using backward model simplification [42] and performed Tukey's honest significance tests to identify significant differences among taxa order.

### Differences of affinity within orders

To characterise the 60 frequent taxa as either specialist, tolerant or avoider of the three land-use types, we calculated their affinities, as detailed in equation 2. For each affinity of every taxon, we computed bootstrap 95% confidence intervals using the bias-corrected accelerated percentile (BCa) method (which allows the construction of non-parametric intervals [43]). For a given taxon and land-use type, a BCa confidence interval of the affinity overlapping with zero means that the affinity with this land-use type was not significantly different from zero, and this taxon was considered tolerant to this land-use type. A taxon with a significant positive affinity with a given land-use type characterised a specialist of this land-use type, while a significant negative affinity characterised an avoider of this land-use type.

## Results

### Dataset collected in 2010

The dataset collected after one year of monitoring covered the whole country thanks to 538 participants (Figure 1A). Among the 283 insect taxa observed, there were 60 frequent and 223 infrequent taxa distributed among the four main insect orders, representing 81% and 19% of the observations, respectively (Table 1; see also Tables S1 & S2 for a precise description of the taxa). Within frequent taxa, Hymenoptera and Diptera were the most frequently observed, followed by Coleoptera and Lepidoptera (Table 1). Infrequent taxa were equally observed among orders, although there were slightly more observations of Lepidoptera (Table 1). In all, 186 taxa were identified at least to the genus level (56% of the pictures) and 118 were identified to species (Table 1). The following results are robust to the exclusion of the 97 taxa whose taxonomy was not resolved at least to the genus level (Tables S4 & S5).

### Contrasted affinities of flower visitors with land-use types

We found significant effects of the order of insects on the three relative land-use indexes (Table 2). This indicated that for each of the three land-use types, there were at least two orders of insects that differ in their mean values of relative land-use index, i.e. their affinities. Regarding urban land-use, Tukey HSD tests indicated that Lepidopterans exhibited a significantly more negative affinity than did Dipterans and Hymenopterans; Coleopterans' and Dipterans' affinities did not differ from each other but did differ from that of Hymenopterans which was close to zero (Figure 2A). Turning to agricultural land-use, the affinities of Coleopterans, Dipterans and Lepidopterans were positive and significantly higher than was the affinity of Hymenopterans, which was close to zero (Figure 2B). Finally, the affinities of Coleopterans and Lepidopterans with natural land-use were positive and were significantly higher than were the affinities of both Dipterans and Hymenopterans, which were close to zero (Figure 2C).

We found significant effects of the frequency of taxa on the relative urban and natural index (Table 2). These indicated that infrequent taxa had a lower affinity with urban land-use and a higher affinity with natural land-use than did frequent taxa (Figure 2A, C).

### Variations in land-use affinities among frequent taxa

Among the 60 frequent taxa, 38 consisted of either specialists or avoiders of at least one of the three land-use types which shows that our technique of analyses was sensitive in characterizing flower visitor affinities (Figure 3). Regarding urban land-use, there were 25 urban avoiders, mostly within Coleopterans, Dipterans and Lepidopterans, and three urban specialists, comprising one Coleopteran and two taxa of cavity nesting bees from the Megachilidae family (Figure 3A and Table S1). In contrast, there were two avoiders of the agricultural land-use type, both Hymenopterans, and 11 specialists that were mostly Coleopterans, Dipterans and Lepidopterans (Figure 3B and Table S1). For the natural land-use, there were eight avoiders, mostly Hymenopterans, and 18 specialists, mostly Coleopterans, Dipterans and Lepidopterans (Figure 3C and Table S1).

Noticeably, for each land-use type, there was a majority of tolerant taxa whose affinity was not different from zero. Adding these tolerant taxa to the specialists of each habitat, there were 35, 58 and 54 taxa that were either tolerant or preferred urban, agricultural and natural land-uses, respectively (Figure 3).

## Discussion

After one year, the Spipoll collected over 7500 standardised observations of flower visitors distributed across a whole country. These data allowed, for the first time, an estimation and comparison of the affinities that a taxonomically broad array of flower visitors have with the three main types of land-use. We showed contrasting patterns among insect orders and important variations in affinities with the different types of land-use within orders of flower visitors. Overall, our results indicate that, despite these substantial variations among and within insect orders, most flower visitor taxa had a negative affinity with urban areas and a positive affinity with agricultural and natural areas. In the following sections, we compare our results to previous studies and discuss how our findings can be used to assess the effects of land-use changes on flower visitor communities. We then emphasise the benefits of monitoring flower visitors through citizen science. Finally, we highlight the implications of our study for flower visitor conservation and management strategies.

### Affinities of flower visitors with the three land-use types

Our results indicate, with the exception of Hymenopterans, flower visitors displayed a negative affinity with urban areas. Such a negative affinity suggests that urbanized areas can only host poor communities of flower visitors. This is in accordance with previous studies showing that urbanisation has a negative impact on the diversity of Coleoptera [15], [44], Diptera [45], [46] and Lepidoptera [15], [47]. We further expand these previous findings to previously unstudied taxonomic groups such as the Nitidulidae and Cerambycidae (Coleoptera), the Empididae and Sepsidae (Diptera) and the Noctuidae (Lepidoptera), all of which are urban avoiders. Regarding Hymenoptera, their overall tolerance and the preference of cavity-nesting bee taxa for urban areas are in accordance with previous studies [48], [49]. Bee surveys in cities, however, indicated a lower diversity than in the regional pool [50], and the lower affinity of infrequent taxa with urban land-use may partially reflect these diversity changes.

Most flower visitors exhibited a positive affinity with agricultural areas, which suggests that this type of land-use hosts diverse flower visitor communities. This finding may seem in disagreement with previous works showing that bee and hoverfly diversity in agricultural areas decreases with increasing distance from semi-natural habitats [9], [17]. However, because these studies focused on agricultural intensification, their definition of semi-natural habitats included many habitats that fall into agricultural land-use following our definition. Our results thus complement the existing literature; while agricultural intensification has strong negative impacts on flower visitors, the agricultural land-use type, as a whole, is still where most flower visitors find their habitats. This is consistent with the view that European agricultural lands were once species rich [51], and stresses the primary importance of agricultural areas for flower visitor conservation.

Flower visitors displayed affinities with natural land-use that were either positive or close to zero, and specialists of this land-use type were the most numerous. Additionally, infrequent taxa had a higher affinity with this land-use than frequent taxa. These results suggest that natural land-use encourages diverse communities of flower visitors and offers habitats particularly suitable for infrequent taxa. In detail, our data suggest that Coleopteran and Lepidopteran infrequent taxa benefit the most from natural land-use (Table 1 & Figure 2C). Hymenoptera as a whole did not display a positive affinity with natural land-use, which may appear surprising. Nevertheless, considering that about half of the recorded Hymenopteran taxa were bees (Tables S1 & S2) and that 59% of collections made in natural areas were located in forests, this result is consistent with the ecology of European bees being open-area species [52].

### Effects of land-use changes on communities

The affinities of flower visitors with land-use types allow characterisation of the various flower-visitors as either specialist, tolerant or avoider of each land-use type. From 2000 to 2006, France has experienced a 3% increase in urban areas, a 0.2% decrease in agricultural areas and a 0.04% decrease in natural areas [53]. In this context, interpreting flower visitor' affinities with urban land-use as indexes of sensitivity to urbanisation, our results suggest that an increase of urban areas is expected to decrease the diversity of Coleoptera, Diptera and Lepidoptera, as approximately half of their taxa were urban avoiders. In particular, the greater sensitivity of butterflies that we found is coherent with a recent hypothesis [21]. Regarding Hymenoptera, urbanisation is expected to mainly affect community composition, as both urban specialists and avoiders were equally present in this order. Nevertheless, there may be more urban avoiders among infrequent Hymenopterans, and the possibility of change in the diversity of this order of flower visitors should not be excluded.

Despite this overall negative impact of urbanisation, 58% of the frequent taxa were either tolerant or specialists of this land-use. This tends to corroborate the idea that urban areas are able to host fairly diverse pollinator communities [54]. In sharp contrast, however, this percentage reached 96% and 90% in agricultural and natural areas, respectively, and our collective results point out that urban communities of flower visitors are merely taxonomically biased subsets of their counterparts in the other two land-use types. As opposed to urbanisation, given that specialist taxa of agricultural and natural land-use were mostly different, conversions between agricultural and natural areas are expected to yield changes in the composition rather than the diversity of flower visitor communities, resulting in typical flower visitor assemblages.

### Monitoring flower visitors with citizen science

Citizen science has demonstrated its efficiency [55], and there are instances dealing with pollinators too [30], [56], [57]. Here we introduced a monitoring scheme that: i) considers all insect flower visitors; ii) collects standardised data that are all validated by entomologists; and iii) aims to provide long-term and country-wide scale indicators on flower visitors. In doing so, the Spipoll should help fulfil the critical need for broad scale knowledge on pollinators, which has been extensively highlighted recently [14], [58], [59]. This monitoring scheme is characterised by an exchange of data for knowledge and results, provided by volunteers and scientists, respectively. Data validation, a crucial step in citizen science [28], is allowed thanks to our photographic approach which also has the advantage to be particularly appealing to participants. Through the Spipoll website, participants can browse the data gathered by the network they are part of, improve their skills in insect and plant identification by using the CAITs or asking for the support of other participants and experts through a forum, and be informed of the latest results obtained from analysing their data. As being involved in the Spipoll benefits both the volunteers and the project, this monitoring scheme is among the good examples of citizen science according to Silvertown [60].

### Management implications

This survey provided information on where flower visitors are and, thus, the areas that conservation and management schemes should focus on. Most flower visitors avoided urban land-use, and this behaviour was stronger for infrequent taxa. Assuming that the 223 infrequent taxa represent a greater number of species than the 60 frequent taxa [61], our results suggest that the diversity of flower visitors in urban areas is lower than in agricultural and natural areas. Thus, although urban parks may be managed to mitigate the effect of urbanisation [62], this strategy may only benefit a highly reduced subset of flower visitors.

Our results suggest that conservation strategies should primarily focus on improving flower visitor habitats within agricultural land-use, as these habitats have the potential to host highly diverse communities of flower-visitors. Management strategies must include practices dedicated to flower visitors [63], and agri-environment schemes, which have been proven effective in enhancing flower visitor diversity [16], [64], [65], should be promoted. Targeting agricultural lands is especially essential, as they occupy more than 40% of the European landscape [66] and have, therefore, a determining role in flower visitor conservation. Additionally, focusing on the agricultural land-use may be a win-win strategy, as crop production may benefit from healthier flower visitor communities [67]. Finally, efforts such as the establishment of the European Union network of protected areas (Natura2000) may help the conservation of flower visitors in natural land-use, especially infrequent visitors. However, an evaluation of the contribution of Natura2000 in protecting flower visitors is needed, as studies regarding other taxa have concluded that this effort has insufficient ability to achieve its major goals of species and habitat protection [68].

In conclusion, by choosing the land-use level for our analyses, we have provided results that complement the existing literature on the effect of habitat degradation within each land-use. The present study highlights the land-use type that should receive attention at a country-wide scale, whereas previous works have identified practices to enhance flower visitor diversity at a local scale. The complementary nature of these findings shall enable policy-makers and managers to implement effective flower visitor conservation and management strategies.

## Supporting Information

Figure S1 Taxonomic resolution of the 556 insect taxa included in the computer-aided identification tool.
(DOC)

Figure S2 Taxonomic resolution of the 333 plant taxa included in the computer-aided identification tool.
(DOC)

Table S1 The description of the 60 frequent taxa.
(PDF)

Table S2 The description of the 223 infrequent taxa.
(PDF)

Table S3 MANOVA results for the three relative land-use indexes.
(DOC)

Table S4 MANOVA results on the 186 taxa resolved at least to the genus level.
(DOC)

Table S5 ANOVA results on the 186 taxa resolved at least to the genus level.
(DOC)
