# Supplementary material for: The Whereabouts of Flower Visitors: Contrasting Land-Use Preferences Revealed by a Country-Wide Survey Based on Citizen Science
Source: PLoS One. 2012 Sep 19;7(9):e45822. doi: 10.1371/journal.pone.0045822 (PMC3446938; doi:10.1371/journal.pone.0045822)
Supplement: Figure S1 — Taxonomic resolution of the 556 insect taxa included in the computer-aided identification tool. (DOC) [file pone.0045822.s001.doc]

**Figure S1.** Taxonomic resolution of the 556 insect taxa included in the computer-aided identification tool (CAIT)

**
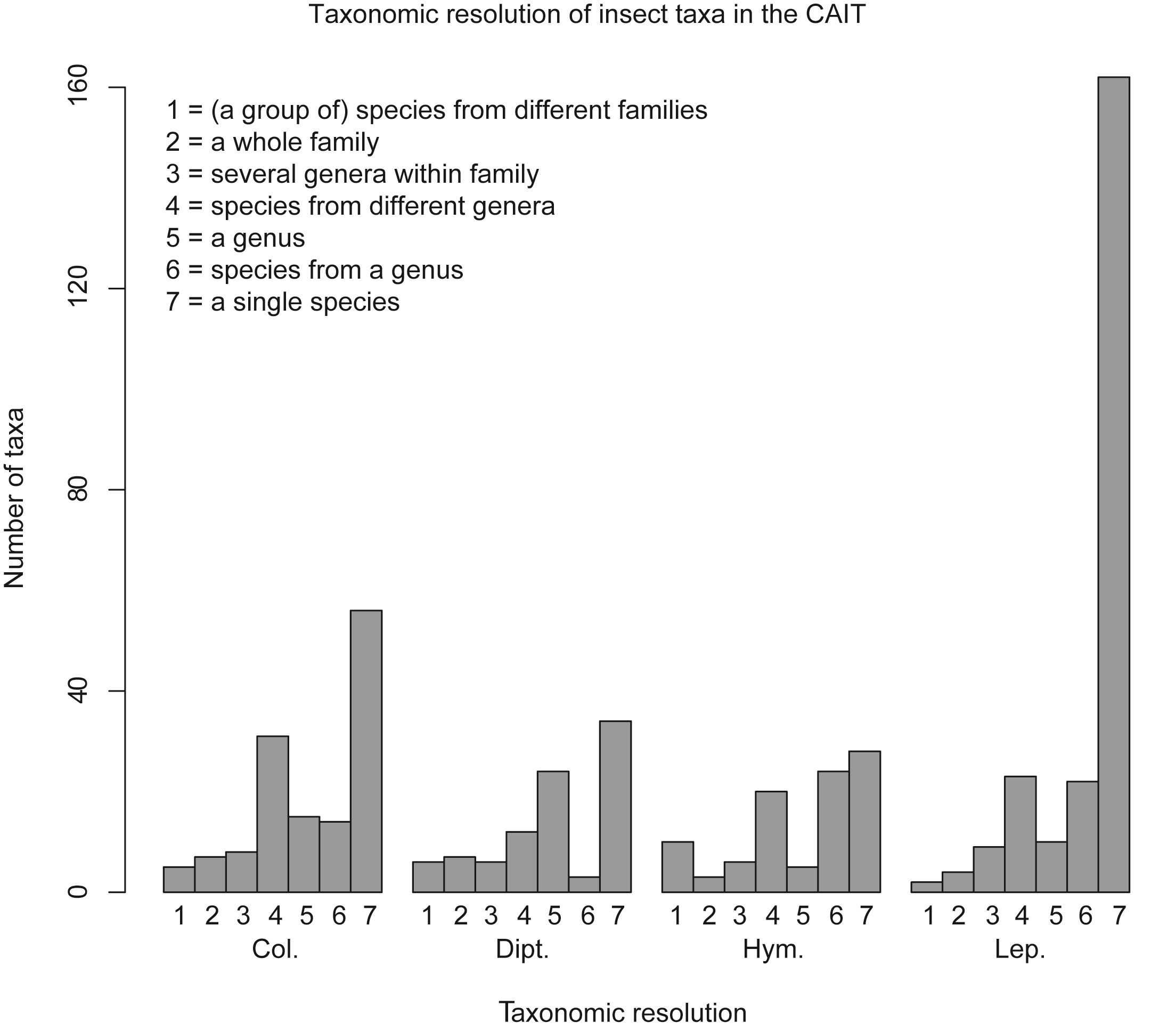
**
