# Supplementary material for: The Whereabouts of Flower Visitors: Contrasting Land-Use Preferences Revealed by a Country-Wide Survey Based on Citizen Science
Source: PLoS One. 2012 Sep 19;7(9):e45822. doi: 10.1371/journal.pone.0045822 (PMC3446938; doi:10.1371/journal.pone.0045822)

**Figure S2.** Taxonomic resolution of the 333 plant taxa included in the computer-aided identification tool (CAIT).


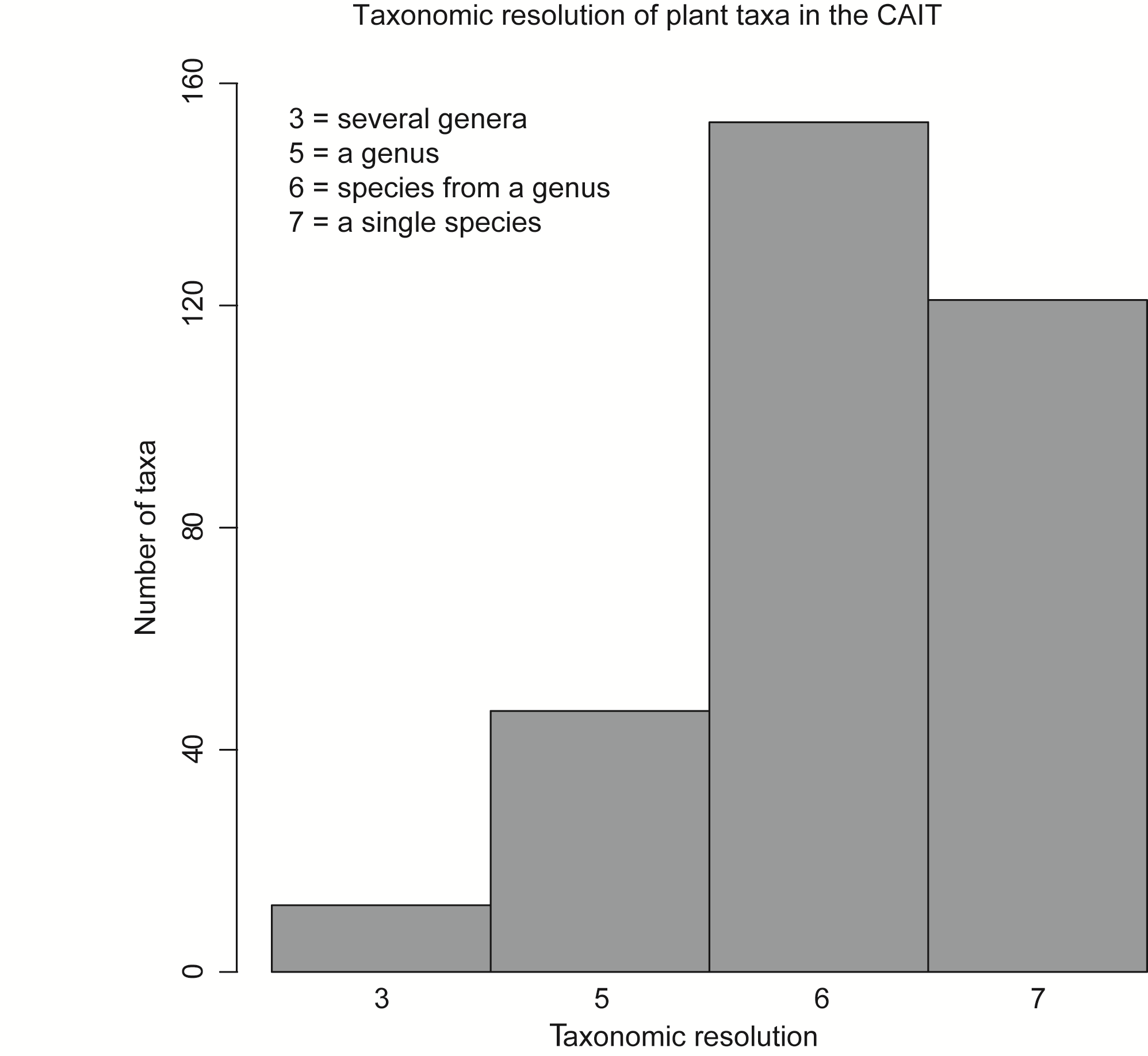

Supplement: Figure S2 — Taxonomic resolution of the 333 plant taxa included in the computer-aided identification tool. (DOC) [file pone.0045822.s002.doc]
