# Supplementary material for: The Whereabouts of Flower Visitors: Contrasting Land-Use Preferences Revealed by a Country-Wide Survey Based on Citizen Science
Source: PLoS One. 2012 Sep 19;7(9):e45822. doi: 10.1371/journal.pone.0045822 (PMC3446938; doi:10.1371/journal.pone.0045822)
Supplement: Table S1 — The description of the 60 frequent taxa. (PDF) [file pone.0045822.s003.pdf]

Table S1. The description of the 60 frequent taxa.

Taxa code	Taxa identity	Species list	Nb. of species included	Taxonomic resolution	Order	Family	Nb. Of pictures	Affinity with land-use types		
								Urb.	Agr.	Nat.
C 1	The banded blister beetles	*Hycleus polymorphus* Pallas, *Mylabris variabilis* Pallas	2	4	Coleoptera	Meloidae	20	-15.74*	5.51	10.71*
C 2	The sap beetles	Nitidulidae spp.	153	2	Coleoptera	Nitidulidae	52	-13.38*	8.98*	3.43
C 3	The spotted longhorn	*Rutpela maculata* Poda	1	7	Coleoptera	Cerambycidae	21	-13.36*	-1.28	14.09*
C 4	The Pachyta and other longhorns	*Acmaeops marginatus* Fabricius, *Acmaeops pratensis* Laicharting, *Acmaeops septentrionis* Thomson, *Anastrangalia dubia* Scopoli, *Anastrangalia reyi* Heyden, *Anastrangalia sanguinolenta* Linnaeus, *Anisarthron barbipes* Schrank, *Anisorus quercus* (female) Goeze, *Brachyta interrogationis* Linnaeus, *Cortodera femorata* Fabricius, *Cortodera humeralis* Schaller, *Grammoptera abdominalis* Stephens, *Grammoptera ruficornis* Fabricius, *Deilus fugax* Olivier, *Leptura aethiops* Poda, *Pachyta quadrimaculata* Linnaeus, *Pachytodes cerambyciformis* Pic, *Pachytodes erraticus* Dalman, *Paracorymbia hybrida* Rey, *Paracorymbia maculicornis* De Geer, *Pedostrangalia pubescens* Fabricius, *Pseudovadonia livida* Fabricius, *Stenocorus meridianus* Linnaeus, *Stenurella nigra* Linnaeus, *Stictoleptura fontenayi* Mulsant, *Stictoleptura rubra* (male) Linnaeus, *Stictoleptura scutellata* Fabricius, *Tetropium castaneum* Linnaeus	28	4	Coleoptera	Cerambycidae	41	-10.98*	-5.01	16.58*
C 5	The seven-spot ladybird	*Coccinella septempunctata* Linnaeus	1	7	Coleoptera	Coccinellidae	80	-10.57*	9.58*	1.64
C 6	The other leaf beetles	*Chrysolina* spp. (except *C. americana* Linnaeus), *Cryptocephalus* spp. (except *C. rugicollis* G.A.Olivier, *C. trimaculatus* Rossi and *C. vittatus* Fabricius), *Entomoscelis* spp., *Oreina* spp.	161	4	Coleoptera	Chrysomelidae	35	-9.87	5.62	5.57
C 7	The tumbling flower beetles	Mordellidae spp.	81	2	Coleoptera	Mordellidae	59	-9.4*	4.28	5.79*
C 8	The metallic chafers with patterns	*Cetonia aurata* Linnaeus, *Cetonia carthami* Gory & Percheron, *Protaetia cuprea* Fabricius, *Protaetia fieberi* Kraatz, *Protaetia lugubris* Herbst	5	4	Coleoptera	Cetoniidae	65	-9.05*	4.2	5.45*
C 9	The dark silky chafers	*Tropinota hirta* Poda, *Tropinota squalida* Lindberg	2	5	Coleoptera	Cetoniidae	19	-7.82	2.31	6.66
C 10	The tawny longhorn	*Paracorymbia fulva* De Geer	1	7	Coleoptera	Cerambycidae	28	-7.18*	-0.18	7.81*
C 11	The white-spotted rose beetle	*Oxythyrea funesta* Poda	1	7	Coleoptera	Cetoniidae	83	-4.36	4.2	0.5

C 12	The black dasytids	*Aplocnemus impressus* Marsham, *Aplocnemus nigricornis* Fabricius, *Dasytes niger* Linnaeus, *Dasytes plumbeus* Müller, *Dasytes obscurus* Gyllenhal, *Enicopus hirtus* Linnaeus	6	4	Coleoptera	Dasytidae	22	-1.14	2.6	-0.55
C 13	The pollen-feeding beetles	*Anogcodes ferrugineus* Schrank, *Anogcodes rufiventris* Scopoli, *Anogcodes ustulatus* Scopoli, *Oedemera femoralis* Olivier, *Oedemera femorata* Scopoli, *Oedemera lateralis* Gebler, *Oedemera lurida* Marsham, *Oedemera monticola* Vihla, *Oedemera nobilis* Scopoli, *Oedemera flavipes* Fabricius, *Oedemera podagrariae* Linnaeus, *Oedemera pthysica* Scopoli, *Oedemera simplex* Linnaeus, *Oedemera tristis* W. Schmidt, *Oedemera virescens* Linnaeus	14	4	Coleoptera	Oedemeridae	205	1.58	1.71	-2.59
C 14	The skin beetles	Dermestidae spp.	81	4	Coleoptera	Dermestidae	45	5.27	8.84*	-13.63*
C 15	The ladybirds	Coccinellidae spp. (except *Coccinella septempunctata* Linnaeus)	110	3	Coleoptera	Coccinellidae	63	8.34*	-3.3	-4.93
D 1		*Rhingia campestris* Meigen, *Rhingia borealis* Ringdahl, *Rhingia rostrata* Linnaeus	3	6	Diptera	Syrphidae	30	-19.37*	10.64*	9.83*
D 2	The tawny tachinids (1)	*Nowickia* spp., *Tachina fera* Linnaeus, *Tachina magnicornis* Zetterstedt	7	4	Diptera	Tachinidae	92	-15.71*	9*	7.05*
D 3	The face fly (male)	*Musca autumnalis* De Geer (male)	1	7	Diptera	Muscidae	33	-13.74*	11.84*	3.04
D 4	The empidids	Empididae spp.	208	2	Diptera	Empididae	57	-13.16*	6.97*	7.19*
D 5	The tawny tachinids (2)	*Ectophasia* spp., *Phasia hemiptera* Fabricius	4	3	Diptera	Tachinidae	47	-11.59*	5.53	5.2
D 6	The thin hoverflies	*Epistrophella* spp., *Melangyna* spp., *Melanostoma* spp., *Meligramma* spp., *Meliscaeva* spp., *Neoascia* spp., *Platycheirus* spp., *Sphaerophoria* spp. (except *S. scripta* Linnaeus), *Syritta* spp. (except *S. pipiens* Linnaeus)	72	4	Diptera	Syrphidae	53	-11.07*	6.18	5.87*
D 7	The black scavenger flies	Sepsidae spp.	24	2	Diptera	Sepsidae	37	-9.82*	5.43	4.99
D 8	The other bee flies	*Bombylius* spp., *Systoechus* spp.	27	3	Diptera	Bombyliidae	54	-9.82*	5.04	5.15
D 9	The cylindric tachinids	*Cylindromyia* spp.	12	5	Diptera	Tachinidae	28	-4.57	3.22	1.69
D 10	The other thick-headed flies	*Melanosoma* spp., *Physocephala* spp., *Sicus* spp., *Thecophora* spp., *Zodion* spp.	21	4	Diptera	Conopidae	30	-4.42	-2.47	7.23
D 11	The other Eristalis hoverflies	*Eristalis* spp. (except *E. intricaria* Linnaeus)	13	4	Diptera	Syrphidae	339	-2.93	1.6	1.33
D 12	The Syrphus hoverflies	*Syrphus* spp.	5	5	Diptera	Syrphidae	74	-2.9	1.39	1.37
D 13	The marmalade hoverfly	*Episyrphus balteatus* De Geer	1	7	Diptera	Syrphidae	184	-1.77	3.33	-1.74

D 14	The other hoverflies	*Dasysyrphus* spp., *Didea* spp., *Epistrophe* spp., *Eupeodes* spp., *Parasyrphus* spp., *Scaeva* spp., *Sericomyia* spp., *Temnostoma* spp. (except *T. bombylans* Fabricius and *T. vespiforme* Linnaeus), *Xanthogramma* spp.	54	4	Diptera	Syrphidae	116	0.5	0.82	-1.63
D 15	The feather holder hoverfly	*Sphaerophoria scripta* Linnaeus	1	7	Diptera	Syrphidae	292	1.1	2.74	-3.22*
D 16	The thick-legged hoverfly	*Syritta pipiens* Linnaeus	1	7	Diptera	Syrphidae	116	1.92	4.5	-5.94*
D 17	The Myathropa florea hoverfly	*Myathropa florea* Linnaeus	1	7	Diptera	Syrphidae	64	2.28	-0.61	-0.98
D 18	The dark stripped flies	*Sarcophaga* spp.	84	5	Diptera	Sarcophagidae	148	3.25	0.44	-3.61
H 1	The black and yellow tenthredinids	*Allantus viennensis* Schrank, *Macrophya montana* Scopoli (female), *Tenthredo* spp. (except *T. colon* Klug, *T. crassa* Scopoli, *T. livida* Linnaeus, *T. velox* Fabricius, *T. solitaria* Scopoli, *T. ferruginea* Schrank, *T. koehleri* Klug, *T. bifasciata* O. F. Müller, *T. caucasica* Eversmann, *T. neobesa* Zombori, *T. campestris* Linnaeus, *T. mesomela* Linnaeus, *T. mioceras* Enslin, *T. obsoleta* Klug, *T. olivacea* Klug)	47	4	Hymenoptera	Tenthredinidae	26	-10.14*	1.56	9.64*
H 2	The ants	Formicidae spp.	218	2	Hymenoptera	Formicidae	141	-3.93	-3.22	6.8*
H 3	The classic wasps	*Dolichovespula* spp., *Polistes* spp., *Vespula* spp. (except *V. rufa* Linnaeus)	18	4	Hymenoptera	Vespidae	159	-2.9	2.23	0.54
H 4	The halictids (Halictus and Lasioglossum) (males)	*Halictus* spp. (males), *Lasioglossum* spp. (males)	119	3	Hymenoptera	Halictidae	187	-1.57	4.95*	-2.69
H 5	The other bumble bees	*Bombus confusus* Schenck, *Bombus hortorum* Linnaeus, *Bombus hypnorum* Linnaeus, *Bombus jonellus* Kirby, *Bombus lapidarius* Linnaeus, *Bombus lucorum* Linnaeus, *Bombus magnus* Vogt, *Bombus pomorum* Panzer, *Bombus pratorum* Linnaeus, *Bombus ruderarius* Müller, *Bombus ruderatus* Fabricius, *Bombus rupestris* Fabricius, *Bombus soroeensis* Fabricius, *Bombus subterraneus* Linnaeus, *Bombus terrestris* Linnaeus, *Bombus wurflenii* Radoszkowski.	16	6	Hymenoptera	Apidae	605	-0.1	1.4	-1.29
H 6	The mason wasps	*Discoelius* spp., *Eumenes* spp., *Ischnogasteroides picteti* Saussure, *Katamenes arbustorum* Panzer, *Leptochilus* spp.	22	3	Hymenoptera	Vespidae	61	0.67	1.43	-2.72
H 7	The honey bee	*Apis mellifera* Linnaeus	1	7	Hymenoptera	Apidae	601	1.31	0.37	-1.94*
H 8	The tawny bumble bee	*Bombus humilis* Illiger, *Bombus muscorum* Linnaeus, *Bombus pascuorum* Scopoli	3	6	Hymenoptera	Apidae	377	1.61	-1.72	0.28
H 9	The halictids (Halictus and Lasioglossum) (females)	*Halictus* spp. (females), *Lasioglossum* spp. (females)	119	3	Hymenoptera	Halictidae	335	1.64	1.68	-3.1*

H 10	The Anthophora bees and alike	*Amegilla* spp., *Anthophora* spp. (except *A. plumipes* Pallas (male)), *Eucera* spp.	68	4	Hymenoptera	Apidae	55	2.75	-4.56	1.19
H 11	The carpenter bees	*Xylocopa valga* Gerstaecker, *Xylocopa violacea* Linnaeus (female)	2	6	Hymenoptera	Apidae	73	5.53	-7.8*	1.92
H 12	The grass-carrying wasp	*Isodontia mexicana* Saussure	1	7	Hymenoptera	Sphecidae	40	6.56	2.01	-8.19*
H 13	The other leafcutter or mason bees	*Megachile* spp. (except *M. lagopoda* Linnaeus (male), *M. maritima* Kirby (male), *M. nigriventris* Schenck (male), *M. willughbiella* Kirby (male)), *Osmia* spp. (except *O. bicolor* Schrank (female), *O. cornuta* Latreille, *O. rufa* Linnaeus)	74	4	Hymenoptera	Megachilidae	129	10.53*	-5.04*	-5.44*
H 14	The potter bees	*Anthidiellum* spp., *Anthidium* (except *A. punctatum* Latreille), *Icteranthidium laterale* Latreille, *Pseudoanthidium* spp., *Stelis signata* Latreille	22	4	Hymenoptera	Megachilidae	75	12.68*	-3.54	-8.19*
L 1	The gatekeepers	*Pyronia tithonus* Linnaeus, *Pyronia cecilia* Vallantin	2	6	Lepidoptera	Nymphalidae	51	-17.98*	13.03*	6.72*
L 2	The small fritillaries and others	*Argynnis adippe* Denis & Schiffermüller, *Argynnis niobe* Linnaeus, *Boloria* spp., *Brenthis* spp.	17	4	Lepidoptera	Nymphalidae	44	-17.96*	2.53	15.78*
L 3	The peacock butterfly	*Aglais io* Linnaeus	1	7	Lepidoptera	Nymphalidae	30	-14.38*	9.11	5.58
L 4	The other owlet moths	Noctuidae spp. (except *Abrostola* spp., *Amata* spp., *Anarta myrtilli* Linnaeus, *Atolmis rubricollis* Linnaeus, *Autographa* spp., *Bena bicolorana* Fuessly, *Callimorpha dominula* Linnaeus, *Euclidia mi* Clerck, *Charanyca trigrammica* Hufnagel, *Chrysodeixis chalcites* Esper, *Ctenoplusia accentifera* Lefebvre, *Cucullia argentea* Hufnagel, *Cybosia mesomella* Linnaeus, *Deltote bankiana* Fabricius, *Diachrysia* spp., *Dichonia aprilina* Linnaeus, *Dicycla oo* Linnaeus, *Drasteria cailino* Lefebvre, *Dysgonia algira* Linnaeus, *Eilema* spp., *Acontia trabealis* Scopoli, *Enterpia laudeti* Boisduval, *Euchalcia* spp., *Euplagia quadripunctaria* Poda, *Gortyna borelii* Pierret, *Grammodes bifasciata* Petagna, *Herminia* spp., *Hypena* spp., *Lamprotes c-aureum* Knoch, *Macdunnoughia confusa* Stephens, *Macrochilo* spp., *Miltochrista miniata* Forster, *Moma alpium* Osbeck, *Noctua fimbriata* Schreber, *Noctua pronuba* Linnaeus, *Panchrysia* spp., *Paracolax* spp., *Phlogophora meticulosa* Linnaeus, *Plusia* spp., *Polychrysia moneta* Fabricius, *Syngrapha* spp., *Thumatha senex* Hübner, *Thysanoplusia* spp., *Trichoplusia ni* Hübner)	533	4	Lepidoptera	Noctuidae	23	-13.55*	4.21	10.65*
L 5	The tawny skippers	*Ochlodes sylvanus* Esper, *Thymelicus acteon* Rottemburg, *Thymelicus lineolus* Ochsenheimer, *Thymelicus sylvestris* Poda	4	3	Lepidoptera	Hesperiidae	44	-12.71*	6.65	6.97*

L 6	The Euphydryas and Melitaea fritillaries	*Euphydryas cynthia* Denis & Schiffermüller (female), *Euphydryas intermedia* Ménétriés, *Euphydryas maturna* Linnaeus, *Melitaea athalia* Rottemburg, *Melitaea aurelia* Nickerl, *Melitaea deione* Geyer, *Melitaea parthenoides* Keferstein, *Melitaea varia* Meyer-Dür	8	4	Lepidoptera	Nymphalidae	37	-12.66*	4.37	9.27*
L 7	The scotch burnet and other burnets	*Zygaena anthyllidis* Boisduval, *Zygaena brizae* Esper, *Zygaena carniolica* Scopoli, *Zygaena contaminei* Boisduval, *Zygaena corsica* Boisduval, *Zygaena cynarae* Esper, *Zygaena ephialtes* Linnaeus, *Zygaena erythrus* Hübner, *Zygaena exulans* Hohenwarth, *Zygaena fausta* Linnaeus, *Zygaena filipendulae* Linnaeus, *Zygaena hilaris* Ochsenheimer, *Zygaena lonicera* Scheven, *Zygaena loti* Denis & Schiffermüller, *Zygaena minos* Denis & Schiffermüller, *Zygaena nevadensis* Rambur, *Zygaena occitanica* Villers, *Zygaena osterodensis* Reiss, *Zygaena purpuralis* Brünnich, *Zygaena romeo* Duponchel, *Zygaena transalpina* Esper, *Zygaena trifolii* Esper, *Zygaena viciae* Denis & Schiffermüller	23	6	Lepidoptera	Zygaenidae	47	-7.97*	-1.33	10.29*
L 8	The meadow brown and others	*Hyponephele lupina* Costa, *Hyponephele lycaon* Kühn, *Maniola jurtina* Linnaeus	3	4	Lepidoptera	Nymphalidae	114	-7.79*	7.7*	1.31
L 9	The hummingbird hawk-moth	*Macroglossum stellatarum* Linnaeus	1	7	Lepidoptera	Sphingidae	70	-5.79	5.01	0.81
L 10	The males of silver-studeed blue and others	*Plebeius argus Linnaeus (male), Plebeius argyrognomon* Bergsträsser (male), *Plebeius idas* Linnaeus (male), *Plebeius pylaon* Fischer von Waldheim (male), *Polyommatus amandus* Schneider (male), *Polyommatus bellargus* Rottemburg (male), *Polyommatus coridon* Poda (male), *Polyommatus dorylas* Denis & Schiffermüller (male), *Polyommatus eros* Ochsenheimer (male), *Polyommatus escheri* Hübner (male), *Polyommatus hispana* Herrich-Schäffer (male), *Polyommatus icarus* Rottemburg (male), *Polyommatus thersites* Cantener (male)	13	4	Lepidoptera	Lycaenidae	48	-5.53	0.83	5.89

L 11	The brown blues	*Cupido alcetas* Hoffmannsegg (female), *Cupido minimus* Fuessly, *Cupido osiris* Meigen (female), *Glaucopsyche iolas* Ochsenheimer (female), *Plebeius agestis* Denis & Schiffermüller, *Plebeius artaxerxes* Fabricius, *Plebeius morronensis* Ribbe, *Plebeius pylaon* Fischer von Waldheim (female), *Polyommatus amandus* Schneider (female), *Polyommatus bellargus* Rottemburg (female), *Polyommatus coridon* Poda (female), *Polyommatus daphnis* Denis & Schiffermüller (female), *Polyommatus dorylas* Denis & Schiffermüller (female), *Polyommatus eros* Ochsenheimer (female), *Polyommatus escheri* Hübner (female), *Polyommatus hispana* Herrich-Schäffer (female), *Polyommatus icarus* Rottemburg (female), *Polyommatus semiargus* Rottemburg (female), *Polyommatus thersites* Cantener (female)	19	4	Lepidoptera	Lycaenidae	43	-2.96	-0.79	4.95
L 12	The cabbage white and others	*Pieris* spp. (except P. *bryoniae* Hübner, *P. napi* Linnaeus)	4	6	Lepidoptera	Pieridae	141	-2.95	5.04*	-1.48
L 13	The scarce swallowtail	*Iphiclides podalirius* Linnaeus	1	7	Lepidoptera	Papilionidae	42	-1.48	-0.28	1.27

The identity of the 60 frequent taxa. “Taxa code” corresponds to the frequent taxa represented in Fig. 3. The taxonomic resolution is coded as 2 = a whole family, 3 = several genera within a family, 4 = species from different genera, 5 = a genus, 6 = species from a genus and 7 = a single species. Affinities with the three land-use types (Urb., Agr. and Nat. standing for Urban, Agricultural and Natural, respectively) marked with a “*” are significantly different from 0 and thus define taxa that are either specialist or avoider of a land-use type.
