# Supplementary material for: The Whereabouts of Flower Visitors: Contrasting Land-Use Preferences Revealed by a Country-Wide Survey Based on Citizen Science
Source: PLoS One. 2012 Sep 19;7(9):e45822. doi: 10.1371/journal.pone.0045822 (PMC3446938; doi:10.1371/journal.pone.0045822)
Supplement: Table S2 — The description of the 223 infrequent taxa. (PDF) [file pone.0045822.s004.pdf]

Table S2. The description of the 223 infrequent taxa.

Taxa identity	Species list	Nb. of species included	Taxonomic resolution	Order	Family	Nb. Of pictures
	Ptinomorphus imperialis Linnaeus	1	7	Coleoptera	Anobiidae	1
The shining buprestid beetles	*Anthaxia* spp. (except *A. confusa* Gory, *A. godeti* Gory & Laporte, *A. helvetica* Stierlin, *A. hungarica* Scopoli, *A. nitidula* Linnaeus (female), *A. millefolii* Fabricius, *A. morio* Fabricius, *A. nigrojubata* Roubal, *A. quadripunctata* Linnaeus, *A. sepulchralis* Fabricius, *A. umbellatarum* Fabricius)	26	4	Coleoptera	Buprestidae	21
The spotted buprestid beetles	*Acmaeodera bipunctata* Olivier, *Acmaeodera degener* Scopoli, *Acmaeodera quadrifasciata* Rossi, *Acmaeoderella* spp.	7	4	Coleoptera	Buprestidae	2
The drab wood-boring beetles	*Acmaeodera cylindrica* Fabricius, *Anthaxia confusa* Gory, *Anthaxia godeti* Gory & Laporte, *Anthaxia helvetica* Stierlin, *Anthaxia hungarica* Scopoli, *Anthaxia millefolii* Fabricius, *Anthaxia morio* Fabricius, *Anthaxia nigrojubata* Roubal, *Anthaxia quadripunctata* Linnaeus, *Anthaxia sepulchralis* Fabricius, *Anthaxia umbellatarum* Fabricius, *Aphanisticus* spp.	17	4	Coleoptera	Buprestidae	2
	Byturus ochraceus Scriba	1	7	Coleoptera	Byturidae	2
The raspberry beetle	*Byturus tomentosus* De Geer	1	7	Coleoptera	Byturidae	2
The Cantharids (1)	*Cantharis fusca* Linnaeus, *Cantharis obscura* Linnaeus, *Cantharis pulicaria* Fabricius, *Cantharis rustica* Fallén	4	6	Coleoptera	Cantharidae	15
	Agapanthia cardui Linnaeus	1	7	Coleoptera	Cerambycidae	1
The flower longhorn with six patches	*Anoplodera sexguttata* Fabricius	1	7	Coleoptera	Cerambycidae	1
The flower longhorn with a black suture	*Stenurella melanura* Linnaeus	1	7	Coleoptera	Cerambycidae	7
The golden flower longhorn	*Leptura aurulenta* Fabricius	1	7	Coleoptera	Cerambycidae	1
The red flower longhorn (female)	*Stictoleptura rubra* Linnaeus (female)	1	7	Coleoptera	Cerambycidae	2
The one-spot flower longhorn	*Vadonia unipunctata* Fabricius	1	7	Coleoptera	Cerambycidae	3
The three-band longhorn	*Chlorophorus trifasciatus* Fabricius	1	7	Coleoptera	Cerambycidae	2
The masked longhorns	*Chlorophorus figuratus* Scopoli, *Chlorophorus sartor* Müller	2	6	Coleoptera	Cerambycidae	4
	Chlorophorus pilosus Forster	1	7	Coleoptera	Cerambycidae	1
	Chlorophorus varius Müller	1	7	Coleoptera	Cerambycidae	2
	Dinoptera collaris Linnaeus	1	7	Coleoptera	Cerambycidae	5
	Alosterna tabacicolor De Geer	1	7	Coleoptera	Cerambycidae	2

The capricorn beetle	*Cerambyx scopolii* Fuessly	1	7	Coleoptera	Cerambycidae	4
The tawny longhorn beetles	*Agapanthia asphodeli* Latreille, *Agapanthia cynarae* Gyllenhal, *Agapanthia dahli* Richter, *Agapanthia kirby* Gyllenhal, *Agapanthia villosoviridescens* De Geer	5	6	Coleoptera	Cerambycidae	2
The wasp longhorn beetles	*Clytus arietis* Linnaeus, *Clytus lama* Mulsant, *Clytus rhamni* Germar, *Clytus tropicus* Panzer, *Cyrtoclytus capra* Germar, *Xylotrechus antilope* Schönherr	6	5	Coleoptera	Cerambycidae	8
The black longhorns with red legs	*Anoplodera rufipes* Schaller, *Paracorymbia stragulata* Germar, *Ropalopus femoratus* Linnaeus, *Ropalopus varini* Bedel	4	4	Coleoptera	Cerambycidae	2
	Stenurella bifasciata Müller, *Stictoleptura cordigera* Fuessly	2	4	Coleoptera	Cerambycidae	14
	Stenopterus spp.	2	5	Coleoptera	Cerambycidae	20
	Valgus hemipterus Linnaeus	1	7	Coleoptera	Cetoniidae	15
	Gnorimus nobilis Linnaeus	1	7	Coleoptera	Cetoniidae	1
The dark hairless chafers	*Protaetia morio* Fabricius, *Protaetia oblonga* Gory & Percheron, *Protaetia opaca* Fabricius	3	6	Coleoptera	Cetoniidae	8
The bee beetles	*Trichius* spp.	3	5	Coleoptera	Cetoniidae	31
The rosemary beetle	*Chrysolina americana* Linnaeus	1	7	Coleoptera	Chrysomelidae	13
The scarlet lily beetle and others	*Exosoma* spp., *Lilioceris lilii* Scopoli	2	4	Coleoptera	Chrysomelidae	4
	Cryptocephalus vittatus Fabricius	1	7	Coleoptera	Chrysomelidae	3
	Donacia spp., *Plateumaris* spp.	27	3	Coleoptera	Chrysomelidae	1
The two coloured leaf beetles	Chrysomelidae spp. (except *Bruchidius* spp., *Bruchus* spp., *Callosobruchus* spp., *Cassida* spp., *Chrysolina* spp., *Clytra laeviusculalytra* Ratzeburg, *Clytra quadripunctata* Linnaeus, *Coptocephala* spp., *Cryptocephalus* spp., *Entomoscelis* spp., *Labidostomis humeralis* Schneider, *Labidostomis taxicornis* Fabricius, *Lachnaia sexpunctata* Scopoli, *Lachnaia tristigma* Lacordaire, *Oreina* spp., *Oulema duftschmidi* Redtenbacher, *Oulema gallaeciana* Heyden, *Oulema melanopus* Linnaeus, *Timarcha* spp.)	583	2	Coleoptera	Chrysomelidae	6
	Oulema duftschmidi Redtenbacher, *Oulema gallaeciana* Heyden, *Oulema melanopus* Linnaeus	3	6	Coleoptera	Chrysomelidae	2
	Clytra laeviusculalytra Ratzeburg, *Clytra quadripunctata* Linnaeus, *Cryptocephalus rugicollis* G.A.Olivier, *Cryptocephalus trimaculatus* Rossi, *Lachnaia sexpunctata* Scopoli, *Lachnaia tristigma* Lacordaire	6	6	Coleoptera	Chrysomelidae	4
	Coptocephala spp.	4	5	Coleoptera	Chrysomelidae	1
	Trichodes octopunctatus Fabricius	1	7	Coleoptera	Cleridae	1
	Trichodes alvearius Fabricius	1	7	Coleoptera	Cleridae	24
	Trichodes apiarius Linnaeus, *Trichodes leucopsideus* Olivier	2	6	Coleoptera	Cleridae	17
The ladybirds larvae	Coccinellidae spp.	111	2	Coleoptera	Coccinellidae	6
The apple blossom weevil and others	*Anthonomus* spp., *Curculio* spp.	25	3	Coleoptera	Curculionidae	1

The Cyphocleonus and Lixus weevils	*Cyphocleonus* spp., *Lixus* spp.	36	3	Coleoptera	Curculionidae	3
	Phyllobius spp., *Polydrusus* spp.	46	3	Coleoptera	Curculionidae	1
	Dascillus cervinus Linnaeus	1	7	Coleoptera	Dascillidae	1
	Psilothrix spp.	2	4	Coleoptera	Dasytidae	5
	Ampedus spp. (except *A. cinnaberinus* Eschscholtz, *A. rufipennis* Stephens, *A. sanguineus* Linnaeus), *Denticollis* spp., *Hemicrepidius* spp., *Limoniscus* spp., *Megapenthes* spp., *Melanotus* spp., *Pheletes* spp.	39	4	Coleoptera	Elateridae	6
	Elateridae spp. (except *Ampedus* spp., *Anostirus castaneus* Linnaeus, *Anostirus purpureus* Poda, *Calambus bipustulatus* Linnaeus, *Denticollis* spp., *Hemicrepidius* spp., *Limoniscus* spp., *Megapenthes* spp., *Melanotus* spp., *Pheletes* spp.)	168	2	Coleoptera	Elateridae	2
	Lycidae spp.	7	2	Coleoptera	Lycidae	1
	Anthocomus fasciatus Linnaeus	1	7	Coleoptera	Malachiidae	1
	Axinotarsus marginalis Laporte de Castelnau, *Axinotarsus pulicarius* Fabricius, *Clanoptilus elegans* Olivier, *Malachius bipustulatus* Linnaeus, *Malachius lusitanicus* Erichson	5	4	Coleoptera	Malachiidae	8
	Ebaeus collaris Erichson, *Ebaeus thoracicus* Fourcroy	2	5	Coleoptera	Malachiidae	1
	Actenodia billbergi Gyllenhal, *Mylabris connata* Rey, *Mylabris flexuosa* Olivier, *Mylabris hieracii* Graells, *Mylabris quadripunctata* Linnaeus, *Mylabris varians* Gyllenhal	6	4	Coleoptera	Meloidae	5
The oil beetles	*Meloe* spp.	15	5	Coleoptera	Meloidae	2
	Mycterus spp.	3	5	Coleoptera	Mycteridae	2
	Chrysanthia geniculata W. Schmidt, *Chrysanthia viridissima* Linnaeus, *Ischnomera caerulea* Linnaeus, *Ischnomera cinerascens* Pandellé in Grénier, *Ischnomera cyanea* Fabricius, *Ischnomera xanthoderes* Mulsant	6	4	Coleoptera	Oedemeridae	8
The cardinal beetle	*Pyrochroa coccinea* Linnaeus	1	7	Coleoptera	Pyrochroidae	1
	Pyrochroa serraticornis Scopoli	1	7	Coleoptera	Pyrochroidae	2
	Hoplia argentea Poda	1	7	Coleoptera	Rutelidae	3
	Hoplia coerulea Drury	1	7	Coleoptera	Rutelidae	1
	Anisoplia austriaca Herbst, *Phyllopertha horticola* Linnaeus	2	4	Coleoptera	Rutelidae	6
	Scraptiidae spp.	30	2	Coleoptera	Scraptiidae	16
	Hymenalia rufipes Fabricius, *Isomira* spp., *Pseudocistela ceramboides* Linnaeus	30	3	Coleoptera	Tenebrionidae	2
The robber flies	*Asilidae* spp. except (*Laphria* spp.)	158	3	Diptera	Asilidae	3
	Bibio hortulanus Linnaeus (female)	1	7	Diptera	Bibionidae	2
	Hemipenthes maura Linnaeus	1	7	Diptera	Bombyliidae	1
	Hemipenthes morio Linnaeus	1	7	Diptera	Bombyliidae	6
	Hemipenthes velutina Meigen	1	7	Diptera	Bombyliidae	2
	Bombylella atra Scopoli	1	7	Diptera	Bombyliidae	1

Villa spp.	21	5	Diptera	Bombyliidae	17
Geron spp.	3	5	Diptera	Bombyliidae	3
Lomatia spp.	5	5	Diptera	Bombyliidae	4
Usia spp.	7	5	Diptera	Bombyliidae	1
Stomorhina lunata Fabricius	1	7	Diptera	Calliphoridae	23
Thaumatomyia spp.	8	5	Diptera	Chloropidae	18
Myopa spp.	12	5	Diptera	Conopidae	1
Culicidae spp.	58	2	Diptera	Culicidae	6
Mesembrina meridiana Linnaeus	1	7	Diptera	Muscidae	2
Graphomya spp.	2	5	Diptera	Muscidae	18
Anthomyia pluvialis Linnaeus, *Anthomyia procellaris* Rondani, *Limnophora maculosa* Meigen, *Limnophora obsignata* Rondani, *Limnophora riparia* Fallén, *Limnophora tigrina* Am Stein	6	4	Diptera	Muscidae	10
Platystoma spp.	6	5	Diptera	Platystomatidae	2
Scatopsidae spp.	52	2	Diptera	Scatopsidae	7
Sciaridae spp.	81	2	Diptera	Sciaridae	6
Chloromyia spp.	2	5	Diptera	Stratiomyidae	9
Odontomyia flavissima Rossi, *Odontomyia limbata* Meigen	2	6	Diptera	Stratiomyidae	1
Stratiomys chamaeleon Linnaeus, *Stratiomys potamida* Meigen	2	5	Diptera	Stratiomyidae	1
Eristalinus taeniops Wiedemann	1	7	Diptera	Syrphidae	7
Chrysogaster solstitialis Fallén	1	7	Diptera	Syrphidae	11
Ferdinandea cuprea Scopoli	1	7	Diptera	Syrphidae	2
Milesia crabroniformis Fabricius	1	7	Diptera	Syrphidae	3
Volucella pellucens Linnaeus	1	7	Diptera	Syrphidae	11
Volucella inanis Linnaeus	1	7	Diptera	Syrphidae	2
Volucella zonaria Poda	1	7	Diptera	Syrphidae	35
Chrysotoxum bicinctum Linnaeus	1	7	Diptera	Syrphidae	1
Riponnensia splendens Meigen	1	7	Diptera	Syrphidae	4
Chrysotoxum spp. (except *Chrysotoxum bicinctum* Linnaeus)	11	5	Diptera	Syrphidae	13
Eristalinus aneneus Scopoli, *Eristalinus sephulchralis* Linnaeus	2	6	Diptera	Syrphidae	7
Helophilus spp. *Parhelophilus* spp.	7	3	Diptera	Syrphidae	27
Arctophila bombiforme Fallén, *Eriozona syrphoides* Fallén, *Eristalis intricaria* Linnaeus, *Cheilosia illustrata* Harris, *Criorhina ranunculi* Panzer, *Merodon clavipes* Fabricius, *Merodon equestris* Fabricius, *Volucella bombylans* Linnaeus	8	4	Diptera	Syrphidae	19
Callicera spp.	6	5	Diptera	Syrphidae	1
Chrysops spp.	9	5	Diptera	Tabanidae	2
Atylotus spp., *Silvius* spp.	9	3	Diptera	Tabanidae	2

	Tabanidae spp. (except *Atylotus* spp., *Chrysops* spp., *Haematopota pluvialis* Linnaeus, *Silvius* spp.)	70	4	Diptera	Tabanidae	5
	Tachina grossa Linnaeus	1	7	Diptera	Tachinidae	6
	Trichopoda pennipes Fabricius	1	7	Diptera	Tachinidae	2
	Gymnosoma spp.	9	5	Diptera	Tachinidae	18
The stiletto flies	Therevidae spp.	25	2	Diptera	Therevidae	2
The crane flies	Tipulidae spp.	139	2	Diptera	Tipulidae	9
The tawny mining bee (female)	*Andrena fulva* Müller (female)	1	7	Hymenoptera	Andrenidae	2
	Andrena morio Brullé	1	7	Hymenoptera	Andrenidae	5
The mining bees with a red and black abdomen	*Andrena florea* Fabricius, *Andrena hattorfiana* Fabricius, *Andrena labiata* Fabricius, *Andrena marginata* Fabricius, *Andrena pellucens* Perez, *Andrena potentillae* Panzer, *Andrena rosae* Panzer, *Andrena sardoa* Lepeletier, *Andrena schencki* Morawitz	9	6	Hymenoptera	Andrenidae	7
The mining bees with black and white fur	*Andrena agilissima* Scopoli, *Andrena apicata* Smith, *Andrena cineraria* Linnaeus, *Andrena cinerea* Brullé, *Andrena vaga* Panzer	5	6	Hymenoptera	Andrenidae	17
The violet carpenter bee (male)	*Xylocopa violacea* Linnaeus (male)	1	7	Hymenoptera	Apidae	7
The blue small carpenter bees	*Ceratina chalcites* Germar, *Ceratina chalybea* Chevrier, *Ceratina cyanea* Kirby	3	6	Hymenoptera	Apidae	14
The black and white cuckoo bees (1)	*Epeolus fallax* Morawitz, *Melecta* spp., *Thyreus* spp.	19	4	Hymenoptera	Apidae	6
The black and white cuckoo bees (2)	*Epeolus* spp. (except *Epeolus fallax* Morawitz)	6	4	Hymenoptera	Apidae	1
The black, yellow and red cuckoo bees	*Epeoloides coecutiens* Fabricius (male), *Nomada* spp.	95	4	Hymenoptera	Apidae	11
The yellow and black bumble bees	*Bombus barbutellus* Kirby, *Bombus campestris* Panzer	2	6	Hymenoptera	Apidae	34
The yellow, black and white bumble bees	*Bombus bohemicus* Seid, *Bombus norvegicus* Sparre-Schneider, *Bombus sylvestris* Lepeletier, *Bombus vestalis* Geoffroy	4	6	Hymenoptera	Apidae	17
The long-horned bees and others (males)	*Eucera* spp. (males), *Tetralonia* spp. (males)	34	4	Hymenoptera	Apidae	13

The patterned stem sawflies	*Calameuta filiformis* Eversmann, *Calameuta haemorrhoidalis* Fabricius (male), *Calameuta idolon* Rossi, *Cephus brachycercus* C. G. Thomson, *Cephus infuscatus* C. G. Thomson, *Cephus lateralis* Konow, *Cephus pygmaeus* Linnaeus, *Cephus spinipes* Panzer, *Hartigia helleri* Taschenberg, *Hartigia linearis* Schrank, *Hartigia nigra* Harris, *Hartigia xanthostoma* Eversmann, *Janus compressus* Fabricius, *Trachelus tabidus* Fabricius, *Trachelus troglodyta* Fabricius	15	4	Hymenoptera	Cephidae	1
The plain stem sawflies	*Calameuta haemorrhoidalis* Fabricius (female), *Calameuta pallipes* Klug, *Cephus nigrinus* C. G. Thomson, *Janus femoratus* Curtis, *Janus luteipes* Lepeletier	5	4	Hymenoptera	Cephidae	1
The ruby tailed wasps	Chrysididae spp.	121	2	Hymenoptera	Chrysididae	15
	Colletes spp.	23	5	Hymenoptera	Colletidae	17
The gasteruptiid wasps	*Gasteruption* spp.	16	5	Hymenoptera	Gasteruptiidae	24
	Coelioxys spp., *Dioxys* spp.	23	3	Hymenoptera	Megachilidae	6
	Dasypoda hirtipes Fabricius (female)	1	7	Hymenoptera	Melittidae	2
The sapygid wasps	Sapygidae spp.	7	2	Hymenoptera	Sapygidae	3
The scoliid wasps with a yellow patch	*Megascolia maculata* Drury (male), *Scolia* spp.	6	4	Hymenoptera	Scoliidae	14
The black and yellow mud dauber	*Sceliphron caementarium* Drury	1	7	Hymenoptera	Sphecidae	4
The Ammophila digger wasps and others	*Ammophila* spp., *Hoplammophila* spp.	10	3	Hymenoptera	Sphecidae	4
The Bembix digger wasps	*Bembix* spp.	8	5	Hymenoptera	Sphecidae	4
	Chilosphex argyrius Brullé (female), *Sphex pruinosus* Germar (female), *Isodontia splendidula* A. Costa (female), *Prionyx lividocinctus* A. Costa (female), *Sphex flavipennis* Fabricius (female), *Sphex funerarius* Gussakovskij (female), *Palmodes occitanicus* Lepeletier & Serville (female), *Palmodes strigulosus* A. Costa (female), *Podalonia affinis* W. Kirby (female), *Podalonia alpina* Kohl (female), *Podalonia fera* Lepeletier (female), *Podalonia hirsuta* Scopoli (female), *Podalonia luffii* Saunders (female), *Podalonia tydei* Le Guillou (female)	14	4	Hymenoptera	Sphecidae	14
	Chilosphex argyrius Brullé (male), *Sphex pruinosus* Germar (male), *Chalybion femoratum* Fabricius (male), *Chalybion flebile* Lepeletier (male), *Isodontia paludosa* Rossi (male), *Podalonia hirsuta* Radoszkowski (male), *Prionyx subfuscatus* Dahlbom (male), *Sphex atropilosus* Kohl (male), *Sphex leuconotus* Brullé (male)	9	4	Hymenoptera	Sphecidae	8
	Athalia rosae Linnaeus	1	7	Hymenoptera	Tenthredinidae	1
	Allantus laticinctus Serville, *Allantus rufocinctus* Retzius, *Allantus calceatus* Klug, *Dolerus* spp., *Macrophya annulata* Geoffroy, *Macrophya blanda* Fabricius, *Macrophya rufipes* Linnaeus (male), *Tenthredo campestris* Linnaeus	52	4	Hymenoptera	Tenthredinidae	10

	Macrophya albicincta Schrank, *Macrophya albipuncta* Fallén, *Macrophya alboannulata* A. Costa, *Macrophya carinthiaca* Klug	4	6	Hymenoptera	Tenthredinidae	3
	Allantus basalis Klug (female), *Allantus cinctus* Linnaeus (female), *Allantus cingillum* Klug (female), *Allantus cingulatus* Scopoli (female), *Allantus togatus* Panzer, *Allantus truncatus* Klug (female), *Tenthredo koehleri* Klug	7	4	Hymenoptera	Tenthredinidae	2
	Aglaostigma aucupariae Klug, *Aglaostigma fulvipes* Scopoli, *Macrophya rufipes* Linnaeus (female), *Tenthredopsis* spp.	14	4	Hymenoptera	Tenthredinidae	2
	Tenthredo mesomela Linnaeus, *Tenthredo mioceras* Enslin, *Tenthredo obsoleta* Klug, *Tenthredo olivacea* Klug	4	6	Hymenoptera	Tenthredinidae	1
	Tiphia femorata Fabricius	1	7	Hymenoptera	Tiphiidae	5
	Meria spp., *Myzinum* spp.	3	3	Hymenoptera	Tiphiidae	1
	Delta unguiculatum Villers	1	7	Hymenoptera	Vespidae	3
The asian hornet	*Vespa velutina* Lepeletier	1	7	Hymenoptera	Vespidae	6
The hornet	*Vespa crabro* Linnaeus	1	7	Hymenoptera	Vespidae	19
	Adelidae spp.	33	4	Lepidoptera	Adelidae	9
	Pyrausta ostrinalis Hübner, *Pyrausta purpuralis* Linnaeus	2	5	Lepidoptera	Crambidae	9
The jersey tiger	*Euplagia quadripunctaria* Poda	1	7	Lepidoptera	Erebidae	7
The scarlet tiger	*Callimorpha dominula* Linnaeus	1	7	Lepidoptera	Erebidae	1
	Eilema spp.	11	5	Lepidoptera	Erebidae	10
	Idaea aureolaria Denis & Schiffermüller	1	7	Lepidoptera	Geometridae	1
The yellow shell	*Camptogramma bilineata* Linnaeus	1	7	Lepidoptera	Geometridae	2
The bloodvein	*Timandra comae* Schmidt	1	7	Lepidoptera	Geometridae	2
The latticed heath	*Chiasmia clathrata* Linnaeus	1	7	Lepidoptera	Geometridae	2
The chimney sweeper	*Odezia atrata* Linnaeus	1	7	Lepidoptera	Geometridae	1
The yellow banded	*Psodos quadrifaria* Sulzer	1	7	Lepidoptera	Geometridae	1
The geometer moths	Geometridae spp. (except *Abraxas sylvata* Scopoli, *Abraxas grossulariata* Linnaeus, *Angerona prunaria* Linnaeus, *Aplocera* spp., *Archiearis parthenias* Linnaeus, *Aspitates gilvaria* Denis & Schiffermüller, *Aspitates ochrearia* Rossi, *Baptria tibiale* Esper, *Boudinotiana notha* Hübner, *Boudinotiana touranginii* Berce, *Camptogramma bilineata* Linnaeus, *Chiasmia clathrata* Linnaeus, *Chloroclystis v-ata* Haworth, *Cidaria fulvata* Forster, *Ennomos* spp., *Eupithecia* spp., *Gagitodes sagittata* Fabricius, *Gymnoscelis rufifasciata* Haworth, *Idaea aureolaria* Denis & Schiffermüller, *Idaea muricata* Hufnagel, *Odezia atrata* Linnaeus, *Opisthograptis luteolata* Linnaeus, *Ourapteryx sambucaria* Linnaeus, *Pasiphila* spp., *Pseudopanthera macularia* Linnaeus, *Psodos quadrifaria* Sulzer, *Selenia* spp., *Siona lineata* Scopoli, *Timandra comae* Schmidt)	459	2	Lepidoptera	Geometridae	4
The silver-spotted skipper	*Hesperia comma* Linnaeus	1	7	Lepidoptera	Hesperiidae	1

The brown skippers	*Carcharodus alceae* Esper, *Carcharodus boeticus* Rambur, *Carcharodus floccifera* Zella, *Carcharodus lavatherae* Esper, *Erynnis tages* Linnaeus, *Muschampia proto* Ochsenheimer, *Pyrgus andromedae* Wallengren, *Pyrgus armoricanus* Oberthür, *Pyrgus bellieri* Oberthür, *Pyrgus cacaliae* Rambur, *Pyrgus carlinae* Rambur, *Pyrgus carthami* Hübner, *Pyrgus cirsii* Rambur, *Pyrgus malvae* Linnaeus, *Pyrgus onopordi* Rambur, *Pyrgus serratulae* Rambur, *Pyrgus warrenensis* Verity, *Spialia sertorius* Hoffmannsegg	18	4	Lepidoptera	Hesperiidae	16
The sooty copper (male)	*Lycaena tityrus* Poda (male)	1	7	Lepidoptera	Lycaenidae	2
The holly blue	*Celastrina argiolus* Linnaeus (female)	1	7	Lepidoptera	Lycaenidae	3
The baton blue	*Pseudophilotes baton* Bergsträsser (male)	1	7	Lepidoptera	Lycaenidae	1
The geranium bronze	*Cacyreus marshalli* Butler	1	7	Lepidoptera	Lycaenidae	9
The small copper	*Lycaena phlaeas* Linnaeus	1	7	Lepidoptera	Lycaenidae	22
The scarce copper	*Lycaena virgaurea* Linnaeus (male)	1	7	Lepidoptera	Lycaenidae	2
The large copper	*Lycaena dispar* Haworth (male)	1	7	Lepidoptera	Lycaenidae	1
The purple-edged copper	*Lycaena hippothoe* Linnaeus (male)	1	7	Lepidoptera	Lycaenidae	1
	Satyrium w-album Knoch	1	7	Lepidoptera	Lycaenidae	1
	Satyrium spini Denis & Schiffermüller	1	7	Lepidoptera	Lycaenidae	2
	Neozephyrus quercus Linnaeus	1	7	Lepidoptera	Lycaenidae	1
	Satyrium pruni Linnaeus	1	7	Lepidoptera	Lycaenidae	2
The males of holly blue and others	*Celastrina argiolus* Linnaeus (male), *Cupido alcetas* Hoffmannsegg (male), *Cupido osiris* Meigen (male), *Glaucopsyche iolas* Ochsenheimer (male), *Polyommatus daphnis* Denis & Schiffermüller (male), *Polyommatus semiargus* Rottemburg (male)	6	4	Lepidoptera	Lycaenidae	13
The long-tailed blue and the Lang's short-tailed blue	*Lampides boeticus* Linnaeus, *Leptotes pirithous* Linnaeus	2	3	Lepidoptera	Lycaenidae	3
The purple-shot copper and others	*Lycaena alciphron* Rottemburg, *Lycaena dispar* Haworth (female), *Lycaena helle* Denis & Schiffermüller (female), *Lycaena hippothoe* Linnaeus (female), *Lycaena tityrus* Poda (female)	5	6	Lepidoptera	Lycaenidae	6
The damon blue and others	*Polyommatus damon* Denis & Schiffermüller, *Polyommatus dolus* Hübner, *Polyommatus ripartii* Freyer	3	6	Lepidoptera	Lycaenidae	2
The sloe hairstreak and others	*Satyrium acaciae* Fabricius, *Satyrium esculi* Hübner, *Satyrium ilicis* Esper	3	6	Lepidoptera	Lycaenidae	13
The green hairstreaks	*Callophrys avis* Chapman, *Callophrys rubi* Linnaeus	2	6	Lepidoptera	Lycaenidae	1
	Micropterix spp.	14	5	Lepidoptera	Micropterigidae	1
The spotted sulphur	*Acontia trabealis* Scopoli	1	7	Lepidoptera	Noctuidae	6

The treble lines	*Charanyca trigrammica* Hufnagel	1	7	Lepidoptera	Noctuidae	1
The silver Y and others	*Abrostola agnorista* Dufay, *Abrostola asclepiadis* Denis & Schiffermüller, *Abrostola tripartita* Hufnagel, *Abrostola triplasia* Linnaeus, *Autographa aemula* Denis & Schiffermüller, *Autographa bractea* Denis & Schiffermüller, *Autographa gamma* Linnaeus, *Autographa jota* Linnaeus, *Autographa pulchrina* Haworth, *Chrysodeixis chalcites* Esper, *Ctenoplusia accentifera* Lefebvre, *Diachrysia chrysitis* Linnaeus, *Diachrysia chryson* Esper, *Diachrysia nadeja* Oberthür, *Diachrysia stenochrysis* Warren, *Euchalcia bellieri* Kirby, *Euchalcia modestoides* Poole, *Euchalcia variabilis* Piller, *Lamprotes c-aureum* Knoch, *Macdunnoughia confusa* Stephens, *Panchrysia aurea* Hübner, *Panchrysia v-argenteum* Esper, *Plusia festucae* Linnaeus, *Plusia putnami* Grote, *Polychrysia moneta* Fabricius, *Syngrapha ain* Hochenwarth, *Syngrapha devergens* Hübner, *Syngrapha hochenwarthi* Hochenwarth, *Syngrapha interrogationis* Linnaeus, *Thysanoplusia circumscripta* Freyer, *Thysanoplusia daubei* Boisduval, *Thysanoplusia orichalcea* Fabricius, *Trichoplusia ni* Hübner	33	3	Lepidoptera	Noctuidae	9
The western marbled white	*Melanargia occitanica* Esper	1	7	Lepidoptera	Nymphalidae	1
The spanish gatekeeper	*Pyronia bathseba* Fabricius	1	7	Lepidoptera	Nymphalidae	2
The painted lady	*Vanessa cardui* Linnaeus	1	7	Lepidoptera	Nymphalidae	13
The european map	*Araschnia levana* Linnaeus	1	7	Lepidoptera	Nymphalidae	5
The glanville fritillary	*Melitaea cinxia* Linnaeus	1	7	Lepidoptera	Nymphalidae	6
The spotted fritillary	*Melitaea didyma* Esper	1	7	Lepidoptera	Nymphalidae	10
The small tortoiseshell	*Aglais urticae* Linnaeus	1	7	Lepidoptera	Nymphalidae	10
The great banded grayling	*Brintesia circe* Fabricius	1	7	Lepidoptera	Nymphalidae	12
The pearly heath and others	*Coenonympha arcania* Linnaeus, *Coenonympha darwiniana* Staudinger, *Coenonympha gardetta* de Prunner	3	6	Lepidoptera	Nymphalidae	4
The marsh fritillary	*Euphydryas aurinia* Rottemburg	1	7	Lepidoptera	Nymphalidae	1
The marbled white	*Melanargia galatha* Linnaeus	1	7	Lepidoptera	Nymphalidae	23
The dusky heath	*Coenonympha dorus* Esper	1	7	Lepidoptera	Nymphalidae	2
The knapweed fritillary	*Melitaea phoebe* Denis & Schiffermüller	1	7	Lepidoptera	Nymphalidae	1
The dark green fritillary	*Argynnis aglaja* Linnaeus	1	7	Lepidoptera	Nymphalidae	1
The dryad	*Minois dryas* Scopoli	1	7	Lepidoptera	Nymphalidae	3
The yellow-spotted ringlet	*Erebia manto* Denis & Schiffermüller	1	7	Lepidoptera	Nymphalidae	1

The queen of Spain fritillary	*Issoria lathonia* Linnaeus	1	7	Lepidoptera	Nymphalidae	7
The commas	*Polygonia* spp.	2	5	Lepidoptera	Nymphalidae	23
The walls	*Lasiommata maera* Linnaeus, *Lasiommata megera* Linnaeus, *Lasiommata petropolitana* Fabricius	3	6	Lepidoptera	Nymphalidae	16
The silver-washed fritillary	*Argynnis paphia* Linnaeus	1	7	Lepidoptera	Nymphalidae	22
The speckled wood	*Pararge aegeria* Linnaeus	1	7	Lepidoptera	Nymphalidae	11
The ringlet	*Aphantopus hyperantus* Linnaeus	1	7	Lepidoptera	Nymphalidae	2
The red admiral	*Vanessa atalanta* Linnaeus	1	7	Lepidoptera	Nymphalidae	34
The false grayling and others	*Arethusana arethusa* Denis & Schiffermüller, *Chazara briseis* Linnaeus, *Hipparchia alcyone* Denis & Schiffermüller, *Hipparchia aristaeus* Bonelli, *Hipparchia fagi* Scopoli, *Hipparchia genava* Fruhstorrfer, *Hipparchia neomiris* Godart, *Hipparchia semele* Linnaeus, *Hipparchia statilinus* Hufnagel, *Satyrus actea* Esper, *Satyrus ferula* Fabricius	11	4	Lepidoptera	Nymphalidae	3
The chestnut heath and others	*Coenonympha glycerion* Borkhausen, *Coenonympha pamphilus* Linnaeus, *Coenonympha tullia* Müller	3	6	Lepidoptera	Nymphalidae	13
The scotch argus and others	*Erebia aethiops* Esper, *Erebia euryale* Esper, *Erebia ligea* Linnaeus	3	6	Lepidoptera	Nymphalidae	2
The false mnestra ringlet and others	*Erebia aethiopella* Hoffmannsegg, *Erebia alberganus* Prunner, *Erebia epiphron* Knoch, *Erebia lefebvrei* Boisduval, *Erebia medusa* Denis & Schiffermüller, *Erebia melampus* Fuessly, *Erebia meolans* Prunner, *Erebia oeme* Hübner, *Erebia scipio* Boisduval, *Erebia sudetica* Staudinger, *Erebia triaria* Prunner	11	4	Lepidoptera	Nymphalidae	1
The white admirals	*Limenitis camilla* Linnaeus, *Limenitis reducta* Staudinger	2	5	Lepidoptera	Nymphalidae	9
The swallowtail	*Papilio machaon* Linnaeus	1	7	Lepidoptera	Papilionidae	14
The cleopatra (male)	*Gonepteryx cleopatra* Linnaeus (male)	1	7	Lepidoptera	Pieridae	1
The black-veined white	*Aporia crataegi* Linnaeus	1	7	Lepidoptera	Pieridae	5
The yellows	*Colias* spp.	5	5	Lepidoptera	Pieridae	18
The cleopatra (female) and the brimstone	*Gonepteryx cleopatra* Linnaeus (female), *Gonepteryx rhamni* Linnaeus	2	6	Lepidoptera	Pieridae	19
The wood whites	*Leptidea* spp.	3	5	Lepidoptera	Pieridae	1
The orange tip and others	*Anthocharis cardamines* Linnaeus (female), *Anthocharis euphenoides* Staudinger (female), *Euchloe* spp., *Pontia* spp.	7	4	Lepidoptera	Pieridae	2
The dark-veined white and the green-veined white	*Pieris bryoniae* Hübner, *Pieris napi* Linnaeus	2	6	Lepidoptera	Pieridae	6
The plume moths	*Pterophoridae* spp.	98	2	Lepidoptera	Pterophoridae	5

The clearwing moths	*Sesiidae* spp.	53	2	Lepidoptera	Sesiidae	14
The broad-bordered bee hawk-moth	*Hemaris fuciformis* Linnaeus	1	7	Lepidoptera	Sphingidae	14
	Thyris fenestrella Scopoli	1	7	Lepidoptera	Thyridae	1
	Zygaena lavandulae Esper	1	7	Lepidoptera	Zygaenidae	1
The common forester and others	*Adscita* spp., *Jordanita* spp.	11	3	Lepidoptera	Zygaenidae	4

The identity of the 223 infrequent taxa. Numbers in the “Taxonomic resolution” correspond to the following: 2 = a whole family, 3 = several genera within a family, 4 = species from different genera, 5 = a genus, 6 = species from a genus and 7 = a single species.
