# Supplementary material for: The Whereabouts of Flower Visitors: Contrasting Land-Use Preferences Revealed by a Country-Wide Survey Based on Citizen Science
Source: PLoS One. 2012 Sep 19;7(9):e45822. doi: 10.1371/journal.pone.0045822 (PMC3446938; doi:10.1371/journal.pone.0045822)
Supplement: Table S3 — MANOVA results for the three relative land-use indexes. (DOC) [file pone.0045822.s005.doc]

**Table S3.** **MANOVA results for the three relative land-use indexes.**

| **Effect** | **Df** | **Wilks' *λ*** | ***F*-value** | ***P*-value** |
| --- | --- | --- | --- | --- |
| Order | 3,18762 | 0.987 | 11.239 | <0.001*** |
| Frequency | 1,7709 | 0.999 | 2.895 | 0.034* |
| Order*Frequency | 3,18762 | 0.998 | 1.565 | 0.12 |

Type-III MANOVA results for the three relative land-use indexes. All three indexes were included in the model as the dependent variables. The independent variables were the order of taxa, the frequency of taxa and their interaction. 'F-value' is the value from F distribution.
