# Supplementary material for: The Whereabouts of Flower Visitors: Contrasting Land-Use Preferences Revealed by a Country-Wide Survey Based on Citizen Science
Source: PLoS One. 2012 Sep 19;7(9):e45822. doi: 10.1371/journal.pone.0045822 (PMC3446938; doi:10.1371/journal.pone.0045822)
Supplement: Table S4 — MANOVA results on the 186 taxa resolved at least to the genus level. (DOC) [file pone.0045822.s006.doc]

**Table S4. MANOVA results on the 186 taxa resolved at least to the genus level.**

| **Effect** | **Df** | **Wilks' *λ*** | ***F*-value** | ***P*-value** |
| --- | --- | --- | --- | --- |
| Order | 3,10570 | 0.988 | 5.753 | <0.001*** |
| Frequency | 1,4343 | 0.999 | 0.408 | 0.747 |
| Order*Frequency | 3,10570 | 0.997 | 1.38 | 0.191 |

Type-III MANOVA results for the three relative land-use indexes and on the 186 taxa whose taxonomy was resolved at least to the genus level. All three indexes were included in the model as the dependent variables. The independent variables were the order of taxa, the frequency of taxa and their interaction. 'F-value' is the value from F distribution.
