# Supplementary material for: The Whereabouts of Flower Visitors: Contrasting Land-Use Preferences Revealed by a Country-Wide Survey Based on Citizen Science
Source: PLoS One. 2012 Sep 19;7(9):e45822. doi: 10.1371/journal.pone.0045822 (PMC3446938; doi:10.1371/journal.pone.0045822)
Supplement: Table S5 — ANOVA results on the 186 taxa resolved at least to the genus level. (DOC) [file pone.0045822.s007.doc]

**Table S5.** **ANOVA results on the 186 taxa resolved at least to the genus level.**

| **Dependent variables** | **Effect** | **Df** | **F value** | **Pr(>F)** |
| --- | --- | --- | --- | --- |
| Relative urban land-use index | Order | 3 | 12.734 | <0.001*** |
|  | Frequency | 1 | 6.201 | 0.013* |
|  |  |  |  |  |
| Relative agricultural land-use index | Order | 3 | 5.342 | 0.001** |
|  |  |  |  |  |
| Relative natural land-use index | Order | 3 | 10.296 | <0.001*** |
|  | Frequency | 1 | 9.932 | 0.002** |

Type-III ANOVA results for the three relative land-use indexes and on the 186 taxa whose taxonomy was resolved at least to the genus level. The results shown are from the minimum adequate models. The 'F-value' is the value from F distribution.
